# Regulation of acetyl-CoA synthetase transcription by the CrbS/R two-component system is conserved in genetically diverse environmental pathogens

**DOI:** 10.1371/journal.pone.0177825

**Published:** 2017-05-18

**Authors:** Kristin Jacob, Anna Rasmussen, Paul Tyler, Mariah M. Servos, Mariame Sylla, Cecilia Prado, Elizabeth Daniele, Josh S. Sharp, Alexandra E. Purdy

**Affiliations:** 1 Department of Biology, Northern Michigan University, Marquette, Michigan, United States of America; 2 Department of Biology, Amherst College, Amherst, Massachusetts, United States of America; Centre National de la Recherche Scientifique, Aix-Marseille Université, FRANCE

## Abstract

The CrbS/R two-component signal transduction system is a conserved regulatory mechanism through which specific Gram-negative bacteria control acetate flux into primary metabolic pathways. CrbS/R governs expression of acetyl-CoA synthase (*acsA*), an enzyme that converts acetate to acetyl-CoA, a metabolite at the nexus of the cell’s most important energy-harvesting and biosynthetic reactions. During infection, bacteria can utilize this system to hijack host acetate metabolism and alter the course of colonization and pathogenesis. In toxigenic strains of *Vibrio cholerae*, CrbS/R-dependent expression of *acsA* is required for virulence in an arthropod model. Here, we investigate the function of the CrbS/R system in *Pseudomonas aeruginosa*, *Pseudomonas entomophila*, and non-toxigenic *V*. *cholerae* strains. We demonstrate that its role in acetate metabolism is conserved; this system regulates expression of the *acsA* gene and is required for growth on acetate as a sole carbon source. As a first step towards describing the mechanism of signaling through this pathway, we identify residues and domains that may be critical for phosphotransfer. We further demonstrate that although CrbS, the putative hybrid sensor kinase, carries both a histidine kinase domain and a receiver domain, the latter is not required for *acsA* transcription. In order to determine whether our findings are relevant to pathogenesis, we tested our strains in a *Drosophila* model of oral infection previously employed for the study of acetate-dependent virulence by *V*. *cholerae*. We show that non-toxigenic *V*. *cholerae* strains lacking CrbS or CrbR are significantly less virulent than are wild-type strains, while *P*. *aeruginosa* and *P*. *entomophila* lacking CrbS or CrbR are fully pathogenic. Together, the data suggest that the CrbS/R system plays a central role in acetate metabolism in *V*. *cholerae*, *P*. *aeruginosa*, and *P*. *entomophila*. However, each microbe’s unique environmental adaptations and pathogenesis strategies may dictate conditions under which CrbS/R-mediated *acs* expression is most critical.

## Introduction

Bacteria use two-component signal transduction systems (TCSs) to respond to changing extracellular conditions and intracellular physiological cues, enabling them to initiate an appropriate pattern of gene expression or protein activity. Pathogens often sense specific molecules, temperature gradients, or other environmental changes in order to regulate expression of virulence factors that facilitate survival in the targeted host. Members of the *Vibrionaceae* and *Pseudomonas* genera carry more than 40 sensor histidine kinases (HKs), molecules that typically initiate these signaling cascades, but their functions during host encounters or survival in the environment are poorly understood.

*Drosophila* is a powerful model system in which to find new bacterial virulence factors, as well as complementary conserved immune defense mechanisms, that may function in human disease [1,2]. A number of human and insect pathogens, including members of both *Pseudomonas* and *Vibrio*, can infect and kill *Drosophila* by secreting a diverse array of proteins, toxins, and small molecules [3–6]. Recently, studies of *V*. *cholerae* infection of *Drosophila* led to the discovery of a novel virulence mechanism that is defined by the removal of acetate from the fly gastrointestinal tract [7]. Acetate is an abundant short-chain fatty acid in the gastrointestinal tract of mammals and insects that is primarily provided to the host by the commensal microbial community [8]. Acetate plays surprisingly important roles in regulating immune function and physiology [9–11]. *V*. *cholerae* depletes acetate by expressing acetyl-CoA synthetase, an enzyme that converts acetate to acetyl-CoA for energy and biosynthesis [7]. Depletion of acetate from the gastrointestinal tract of the fly causes intestinal steatosis, an inappropriate storage of fats in the fly enterocytes, which facilitates fly mortality [7]. *Acs* transcription is regulated by the CrbS/R TCS, and expression of *acsA*, *crbR*, and *crbS* are all required for *V*. *cholerae* virulence towards *Drosophila* [7]. This mechanism was discovered and characterized in a pandemic strain of *V*. *cholerae* of the O139 serotype that carries both the cholera toxin and toxin-coregulated pilus genes required for causing cholera. However, this TCS is well conserved in sequenced *V*. *cholerae* strains, including environmental, non-toxigenic *V*. *cholerae* isolates. Beyond *V*. *cholerae*, this TCS is widely conserved amongst members of the Vibrionaceae, as well as other gamma-proteobacteria. In this study, we examine the function of this system in environmental strains of *V*. *cholerae* [12], as well as in two members of the *Pseudomonas* genus, *P*. *aeruginosa* and *P*. *entomophila*. *P*. *entomophila* is a natural pathogen of insects [13], and *P*. *aeruginosa* can infect humans as well as a variety of other hosts in the environment [6].

In *V*. *cholerae*, CrbS, an orphan sensor HK, and CrbR, a response regulator, are required for *acs* expression and are thought to comprise a TCS [7]. CrbS is a hybrid HK that consists of a 13–transmembrane pass transporter domain of unknown function with similarity to sodium-solute symporters, a Per-Arndt-Sim domain, a STAC domain [14], a catalytic and ATPase domain, a His-containing phosphoacceptor (HisKA) domain, and a receiver (REC) domain. The homolog of CrbS in *P*. *aeruginosa*, MxtR, was discovered in a transposon mutagenesis screen for mutants that no longer respond to the interbacterial signaling molecule 2-alkyl-4(1*H*)-quinolone [15]. MxtR was hypothesized to function as a redox-responsive signaling molecule that controls gene expression via a LysR transcription factor, MexT [15]. However, a role for MxtR in regulation of *acs* has not been uncovered. CrbS is also homologous to CbrA, an HK of similar structure that regulates metabolism and virulence in *Pseudomonas* strains [16–18], although CbrA is missing the terminal REC domain. Homologs of CrbR have been identified in *P*. *aeruginosa* and *Vibrio vulnificus* as well. The *P*. *aeruginosa* homolog of CrbR, ErdR, is required for regulation of *acs* and ethanol detoxification [19]. The homolog of CrbR in *V*. *vulnificus*, AcsR, directly regulates expression of *acs* [20]. A further link between CrbS, CrbR, and Acs was revealed in a genome-wide study of fitness under different growth conditions in *Shewanella oneidensis*, in which a role for regulation of *acs* was hypothesized [21]. To our knowledge, a signaling pathway that links the CrbS and CrbR proteins has not been defined in any *Pseudomonas* species, and homologs of these genes have not been studied in *P*. *entomophila*.

In this work, we demonstrate that the function of the CrbR and CrbS homologs to regulate *acs* expression, and thus confer the ability to grow on acetate as a sole carbon source, is conserved in diverse strains of *V*. *cholerae* as well as in the human pathogen *P*. *aeruginosa* and the insect pathogen *P*. *entomophila*. However, virulence of *Pseudomonas* towards flies does not appear to involve metabolic regulation through activation of *acs* and uptake of acetate, and these genes do not contribute to the virulence repertoire required for infection of *Drosophila* by *P*. *aeruginosa* or *P*. *entomophila*. These results provide evidence that the CrbS/R two-component signal transduction mechanism, and one of its target genes, is widely conserved in host-associated bacterial genera, but may play different roles in the ecology and virulence of these bacterial groups.

## Materials and methods

### Bacterial strains, fly strains, and media

*P*. *entomophila* L48, *P*. *aeruginosa* PAO1, and *V*. *cholerae* SIO [12] were used as the wild-type parental strains in this study. All strains included in this study are listed in Table 1, and all plasmids are listed in Table 2. The primers used in this study are listed in S1 Table.

**Table 1:** Bacterial strains used in this study.

*Vibrio cholerae*	Description	Reference
**AP94 **	*V*. *cholerae* strain SIO wild-type, Amp^R^	[12]
**AP27 **	SIO Δ*crbS*, Amp^R^	This study
**AP218 **	SIO Δ*acs-1*, Amp^R^	This study
**AP360 **	SIO *crbS*ΔREC, Amp^R^	This study
**AP1661**	SIO *crbS*H798A, Amp^R^	This study
**AP1664**	SIO *crbS*H798Q, Amp^R^	This study
**AP1669**	SIO *crbS*D1081A, Amp^R^	This study
**AP456 **	SIO Δ*crbR*, Amp^R^	This study
**AP1014 **	SIO *crbR*ΔREC, Amp^R^	This study
**AP462 **	SIO/pBBR*lux*, Amp^R^,Cm^R^	This study
**AP431 **	SIO/pPT002 (pBBR*lux*::P_*acs-*SIO_), Amp^R^, Cm^R^	This study
**AP336 **	SIO Δ*crbS*/pPT002 (pBBR*lux*::P_*acs-*SIO_), Amp^R^, Cm^R^	This study
**AP384 **	SIO *crbS*ΔREC/pPT002 (pBBR*lux*::P_*acs*-SIO_), Amp^R^, Cm^R^	This study
**AP1026 **	SIO Δ*crbR*/pPT002 (pBBR*lux*::P_*acs*-SIO_), Amp^R^, Cm^R^	This study
**AP1028 **	SIO *crbR*ΔREC/pPT002 (pBBR*lux*::P_*acs*-SIO_), Amp^R^, Cm^R^	This study
**AP1694**	SIO *crbS*H798A/pPT002 (pBBR*lux*::P_*acs-*SIO_), Amp^R^, Cm^R^	This study
**AP1695**	SIO *crbS*H798Q/pPT002 (pBBR*lux*::P_*acs-*SIO_), Amp^R^, Cm^R^	This study
**AP1696**	SIO *crbS*D1081A/pPT002 (pBBR*lux*::P_*acs-*SIO_), Amp^R^, Cm^R^	This study
***Escherichia coli***		
**S17-1λ*pir***	RP4-2(Km::Tn7,Tc::Mu-1), pro-82, LAMpir, *recA1 endA1 thiE1 hsdR17 creC510 *	[22]
**SM10λ*pir***	*thi thr leu tonA lacY supE recA*::RP4-2-Tc::Mu Km*λpir*	[23]
**DH5αλ*pir***	F^-^ Δ(*lacZYA*‐*argF*)*U169 recA1 endA1 hsdR17 supE44 thi-1 gyrA96 relA1* λ::pir	[24]
**MFD*pir***	MG1655 RP4‐2-Tc::[ΔMu1::*aac(3)IV‐*Δ*aphA*-Δ*nic35*-Δ*Mu2*::*zeo*] Δ*dapA*::(*erm*Δ*pir*) Δ*recA*. Apra^R^, Zeo^R^, Erm^R^	[25]
**AP15 **	MFD*pir*/pAR001 (pHC001B::Δ*crbS*, SIO insert, Km^R^)	This study
**AP207 **	MFD*pir*/pMS001 (pHC001B::Δ*acs-1*, SIO insert, Km^R^)	This study
**AP437 **	MFD*pir*/pED002 (pHC001B::Δ*crbR*, SIO insert, Km^R^)	This study
**AP344 **	MFD*pir*/pPT007 (pHC001B::*crbS*ΔREC, SIO insert, Km^R^)	This study
**AP996 **	MFD*pir*/pED003 (pHC001B::*crbR*ΔREC, SIO insert, Km^R^)	This study
**AP272 **	S17-1λ*pir*/pBBR*lux*,Cm^R^	This study
**AP279 **	S17-1λ*pir*/pPT002(pBBR*lux*::P_*acs*-SIO_), Cm^R^	This study
***Pseudomonas aeruginosa ***		
**PAO1 **	Wild-type	[26]
**PAO1Δ*gacA***	PAO1 ΔPA2586	This study
**PAO1Δ*crbS***	PAO1 ΔPA3271	This study
**PAO1Δ*erdR***	PAO1 ΔPA3604	This study
**PAO1*crbS*ΔREC**	PAO1 PA3271Δnucleotides 3124–3468	This study
**PAO1*erdR*ΔREC**	PAO1 PA3604Δnucleotides19-368	This study
***Pseudomonas entomophila***		
**L48 **	Wild-type	[13]
**L48Δ*crbS***	L48ΔPSEEN1405	This study
**L48Δ*crbR***	L48ΔPSEEN4122	This study
**L48*crbS*ΔREC**	L48 PSEEN1405Δnucleotides 3118–3462	This study
**L48*crbR*ΔREC**	L48 PSEEN4122Δnucleotides 19–369	This study
**L48Δ*acsA***	L48ΔPSEEN3888	This study

**Table 2:** Plasmids used in this study.

**Plasmid**	** Description**	** Reference**
**pBBR*lux***	Reporter gene fusion/cloning vector, Cm^R^	[27]
**pHC001B **	Conjugating vector; Kan^r^, λ*pir*-dependent ori	[28]
**pAR001 **	pHC001B::Δ*crbS*, SIO insert, Km^R^	This study
**pPT002 **	pBBR*lux*::P_*acs-*SIO_, SIO-derived 660bp insert, Cm^R^	This study
**pMMS001 **	pHC001B::Δ*acs-1*, SIO insert, Km^R^	This study
**pED002 **	pHC001B::Δ*crbR*, SIO insert, Km^R^	This study
**pED003 **	pHC001B::*crbR*ΔREC, SIO insert, Km^R^	This study
**pPSV38 **	*Pseudomonas* protein expression vector, Gm^R^	[29]
**pPSV38-*erdR***	*P*. *aeruginosa* ErdR expression vector, Gm^R^	This study
**pPSV38-*crbR***	*P*. *entomophila* CrbR expression vector, Gm^R^	This study
**pEXG2**	Allelic exchange vector for constructing in-frame gene deletions in *Pseudomonas*, Gm^R^	[30]
**pEX-ΔPA3271 **	pEXG2::Δ*crbS*, Gm^R^	This study
**pEX-ΔPA3604 **	pEXG2::Δ*erdR*, Gm^R^	This study
**pEX-ΔPSEEN1405 **	pEXG2::Δ*crbS*, Gm^R^	This study
**pEX-ΔPSEEN4122 **	pEXG2::Δ*crbR*, Gm^R^	This study
**pEX-ΔPA3271REC **	pEXG2::*crbS*ΔREC, Gm^R^	This study
**pEX-ΔPA3604REC **	pEXG2::*erdR*ΔREC, Gm^R^	This study
**pEX-ΔPSEEN1405REC **	pEXG2::*crbS*ΔREC, Gm^R^	This study
**pEX-ΔPSEEN4122REC **	pEXG2::*crbR*ΔREC, Gm^R^	This study
**pEX-ΔPSEEN3888 **	pEXG2::Δ*acsA*, Gm^R^	This study

*P*. *aeruginosa*, *Escherichia coli*, and *V*. *cholerae* strains were cultured in Luria Bertani Miller (LBM) media (Fisher Scientific). LBM agar plates were made by adding 15 g/L agar (Fisher Scientific) to LBM media. *P*. *entomophila* strains were cultured in modified, low-salt LBM media (LBMLS): 10 g peptone, 5 g yeast extract, 3 g NaCl. LBMLS plates were made by adding 15 g/L agar to LBMLS media.

Minimal media experiments utilized M63 minimal media (VWR) with pH adjusted to 7.0 using NaOH. After autoclaving, 1 mL of 1M MgSO_4_ was added per liter of media. The M63 minimal media was supplemented with 5mM sodium acetate and 5mM glucose as needed.

When antibiotic selection was required, the appropriate media was supplemented with 15 μg/mL gentamicin (VWR) (for *E*. *coli*), 30 μg/mL gentamicin (for *Pseudomonas* spp.), or 100 μg/mL kanamycin (Sigma) (for *V*. *cholerae* and *E*. *coli*). For experiments requiring expression of CrbR or ErdR in *Pseudomonas* species, isopropyl β-D-1-thiogalactopyranoside (IPTG) (GoldBio) was added to the growth media to a final concentration of 1mM to induce expression of these genes from the pPSV38 expression vectors.

All plasmid manipulations were performed in *E*. *coli* DH5α, or DH5αλ*pir*. *E*. *coli* SM10 was utilized to transfer plasmids into *P*. *entomophila* and *P*. *aeruginosa* by conjugation, and *E*. *coli* MFD*pir* cells [31] were used for conjugations into *V*. *cholerae*. Mutant strains constructed for this study were the following: *P*. *entomophila* Δ*acsA*, *P*. *entomophila* ΔPSEEN1405 (*crbS*), *P*. *entomophila* ΔPSEEN4122 (*crbR*), *P*. *entomophila* PSEEN1405ΔREC, *P*. *entomophila* PSEEN4122Δ ΔREC, *P*. *aeruginosa* PAO1 ΔPA3271 (*mxtR*), *P*. *aeruginosa* PAO1 ΔPA3604 (*erdR*), *P*. *aeruginosa* PAO1 *mxtR*ΔREC, and *P*. *aeruginosa* PAO1 *erdR*ΔREC.

### *Pseudomonas* methods

#### Construction of two-component system deletion mutants

TCS gene deletions were constructed as follows: Mutants were constructed from parental strains *P*. *aeruginosa* PAO1 or *P*. *entomophila* L48 by allelic exchange. *E*. *coli* SM10 was utilized to conjugate a pEXG2 allelic exchange vector into *Pseudomonas* spp. pEXG2 plasmids containing desired deletion constructs were conjugated into *P*. *entomophila* using *E*. *coli* SM10, essentially as described by Castang et al. [30]. Deletion constructs for the *acsA*, PSEEN1405, and PSEEN4122 genes were generated by amplifying 1-kb regions flanking the gene to be deleted by polymerase chain reaction (PCR) (KOD Xtreme Kit, EMD Millipore) and then splicing the flanking regions together by overlap extension PCR. *acsA* PCR products contained a 5′ BamHI site and a 3′ KpnI site, PSEEN1405 PCR products contained a 5′ HindIII and a 3′ EcoRI site, and PSEEN4122 PCR products contained a 5′ HindIII and a 3′ BamHI site for cloning into pEXG2. The resulting PCR products were cloned into plasmid pEXG2 [30], yielding plasmids pEX-Δ*acsA*, pEX-ΔPSEEN1405, and pEX-ΔPSEEN4122. These plasmids were then used to create strains *P*. *entomophila* Δ*acsA*, Δ*crbS*, and Δ*crbR*, containing in-frame deletions of the *acsA*, PSEEN1405, and PSEEN4122 genes respectively. The allelic exchange was performed essentially as described by Castang et al. [30]. Target gene deletions were confirmed by colony PCR. For deleting the *crbS* and *crbR* homologs in *P*. *aeruginosa*, a similar protocol was utilized. Both the *mxtR* PCR products and PA3604 PCR products contained a 5′ BamHI site and a 3′ EcoRI site for cloning into the pEXG2 allelic exchange vector. This generated plasmids pEX-Δ*mxtR* and pEX-ΔPA3604, which were utilized to create strains *P*. *aeruginosa* Δ*mxtR* and *P*. *aeruginosa* Δ*erdR*, respectively.

#### Construction of two-component system REC domain mutants

*P*. *entomophila* and *P*. *aeruginosa* mutants were constructed by deleting the REC domain of either the sensor kinase or the response regulator of the CrbS/R TCS. Mutants were constructed from either parental strain *P*. *entomophila* L48 or *P*. *aeruginosa* PAO1 through allelic exchange, as previously described. The *P*. *entomophila* mutants had deletions of nucleotides 3118 to 3462 in the sensor kinase (PSEEN1405), or of nucleotides 19 to 369 in the response regulator (PSEEN4122). *P*. *aeruginosa* mutants had deletions of nucleotides 3124 to 3468 in the sensor kinase (*mxtR*) or of nucleotides 19 to 368 in the response regulator (*erdR*).

#### Construction of expression plasmids encoding *crbR* and *erdR*

The *crbR* gene (PSEEN4122) was amplified by PCR from *P*. *entomophila* L48 chromosomal DNA. This gene was PCR-amplified to contain a 5′ EcoRI restriction enzyme site and a 3′ HindIII restriction enzyme site. The PCR-amplified *crbR* gene was digested with EcoRI and HindIII and ligated into the pPSV38 plasmid expression vector digested with the restriction enzymes described by Rietsch et al. [29]. Plasmid pPSV38 is a derivative of pPSV35 [29] that contains the IPTG-inducible *lacUV5* promoter flanked by two *lac* operators. Plasmid pPSV38-*crbR* (pCrbR) drives the expression of the 4122 gene (PSEEN4122) from *P*. *entomophila* strain L48, under the control of the IPTG-inducible *lacUV5* promoter, and confers resistance to gentamicin. Identical methods were utilized for generating the expression plasmid pPSV38-*erdR* (pErdR), though this gene was amplified by PCR from *P*. *aeruginosa* PAO1 chromosomal DNA.

Relevant sequences of all plasmids used in this study were confirmed by DNA sequencing (Genewiz, South Plainfield, NJ).

#### Plasmid-based expression of *crbR* and *erdR*, RNA extraction, and cDNA synthesis

Wild-type or mutant strains of *P*. *entomophila* or *P*. *aeruginosa* were grown in 5 mL of LBMLS or LBM media, respectively, overnight with shaking at 200 rpm at 30°C or 37°C, respectively. Overnight cultures were used to make electrocompetent cells as described by Choi and Schweizer [32]. Forty microliters of electrocompetent cells were transferred to an electroporation cuvette (USA Scientific). One microliter of either the pPSV38 plasmid, pPSV38-*crbR* plasmid, or pPSV38-*erdR* plasmid was added to the appropriate electrocompetent cells. The *Pseudomonas* strains were transformed as described [32]. The transformed cells were plated on an LBM or LBMLS agar containing 30 μg/mL gentamicin. *P*. *entomophila* cultures were incubated at 30°C and *P*. *aeruginosa* cultures were incubated at 37°C. A single colony from each *P*. *entomophila* culture was used to inoculate 5 mL of LBMLS broth containing 30 μg/mL gentamicin. The resulting cultures were incubated for 16 hours in a 30°C incubator with agitation at 200 rpm. These overnight cultures were used to inoculate 25 mL of LBM or LBMLS broth containing 30 μg/mL gentamicin and 1mM IPTG to a starting OD600nm of 0.03. *P*. *entomophila* cultures were grown at 30°C on a shaker (USA Scientific) set at 200 rpm until an OD600nm of approximately 0.5 was achieved. *P*. *aeruginosa* cultures were processed the same way except that they were grown in LBM media and incubated at 37°C. After the desired absorbance was reached, 10 mL of each culture was transferred to a 15 mL centrifuge tube (VWR) and centrifuged at 3220 *g* (Eppendorf 5810 R) for 10 minutes at 4°C. Cell pellets were then resuspended in 1 mL of RNAzol (Molecular Research Center) and incubated at 60°C for 10 minutes. RNA isolation was conducted essentially as described by Goldman et al. [33]. cDNA synthesis was conducted essentially as described by Wolfgang et al. [34].

#### Quantitative real-time PCR to evaluate transcript abundance

RNA was isolated from wild-type and mutant strains of *P*. *entomophila* and *P*. *aeruginosa* essentially as described by Goldman et al. [33]. Extracted RNA was used for cDNA synthesis essentially as described by Wolfgang et al. [34]. A Nano-Drop 200c spectrophotometer (ThermoFisher) was used to check the concentration and purity of the synthesized cDNA. The abundance of target transcripts relative to *clpX* transcripts was measured by quantitative real-time PCR (qRT-PCR) using the iTaq SYBR Green kit (Bio-Rad) and MyIQ Single-Color Real-Time PCR Detection System (Bio-Rad). Transcript expression data were determined utilizing the ΔΔCt method as described by Livak and Schmittgen [35]. Experiments were performed in duplicate. Real-time PCR primers were tested for amplification efficiency. Only those primer sets that generated a single amplicon, and that had amplification efficiencies greater than or equal to 90% with R^2^ values of 0.9 or higher, were utilized for quantification of gene expression.

#### Role of *acsA* in acetate metabolism utilizing M63 minimal media

The ability of *P*. *entomophila* and *P*. *aeruginosa* TCS mutants and a *P*. *entomophila acsA* mutant to utilize acetate as a sole carbon source was analyzed using M63 minimal media (VWR) supplemented with 5mM sodium acetate (VWR), which we will refer to as M63-acetate broth. As a control, growth assays were also performed utilizing M63 minimal media supplemented with 5mM sodium acetate and 5mM glucose (VWR), which will be referred to as M63-acetate/glucose broth. Growth assays were performed as follows: wild-type *P*. *entomophila*; *P*. *entomophila* mutants Δ*acsA*, Δ*crbS*, and Δ*crbR*; and *P*. *entomophila* REC domain mutants were transformed with either pPSV38 or a plasmid expressing the response regulator (pPSV38-*crbR*), as previously described. Following transformation, a single colony of each transformed strain was inoculated into 25 mL of either M63-acetate or M63-acetate/glucose broth supplemented with 1mM IPTG and 30 μg/mL gentamicin. Cultures were incubated at 30°C with agitation at 200 rpm. Growth was monitored by measuring the OD_600nm_ over a period of 28 hours utilizing a spectrophotometer (Spectronic 20 Genesys). Experiments with wild-type *P*. *aeruginosa*, *P*. *aeruginosa* mutants Δ*mxtR* and Δ*erdR*, and *P*. *aeruginosa* REC domain mutants were performed as described above for *P*. *entomophila*, with the following modifications: *P*. *aeruginosa* strains were transformed with pPSV38 or pPSV38-*erdR*, and cultures were incubated at 37°C. These experiments were repeated twice with duplicate cultures.

### *V*. *cholerae* methods

#### Two-component system deletion mutants and REC domain deletion mutants

Homologs of the VC0303 (*crbS*) and VC2702 (*crbR*) genes were identified in a draft version of the *V*. *cholerae* SIO genome (now published in [36]), and all in-frame deletions were constructed via allelic exchange. To delete the VC0303 gene, splicing by overlap extension (SOE) PCR was used to construct a DNA segment that carried approximately 1000 bp of DNA both upstream and downstream of VC0303, while removing all but 54 base pairs at the 5′ end, and 48 base pairs at the 3′ end, of the gene. The upstream and downstream fragments were amplified using the AR01 and AR02 primers and the AR03 and AR04 primers, respectively, from genomic DNA isolated via the Wizard genomic DNA isolation kit (Promega). The AR02 and AR03 primers carry a complementary 18-bp tag that allows for self-annealing during SOE PCR. The PCR was performed using the High Fidelity PCR SuperMix (Invitrogen), and the resulting product was gel-purified, TA-cloned into pCR2.1-TOPO, and transformed into TOP10 *E*. *coli* cells (Invitrogen). Plasmids carrying inserts of the correct size were verified by sequencing (Genewiz, Cambridge, MA). The plasmids were then digested with *Xho*I and *Spe*I, and the insert was ligated into pHC001B, a derivative of pWM91 that carries a kanamycin resistance gene [28], with T4 DNA ligase (NEB). The ligation reactions were transformed into *E*. *coli* DH5-αλpir for verification. Plasmids carrying correctly-sized inserts were then transformed into MFD*pir* [31], and conjugated into *V*. *cholerae*. Single recombinants were selected on kanamycin and 2,6-diaminopimelic acid (0.3mM), and double recombinants were selected on sucrose plates (10% sucrose, 0.5% yeast extract, 1% tryptone, 1.5% agar (w/v)). *V*. *cholerae* clones carrying the mutant allele were verified by PCR. Deletion of the VC0303 REC domain was performed via the same method, except that primers PT22 and PT35 amplified the upstream sequence from SIO, and the primers PT36 and PT25 amplified the downstream sequence from SIO. Instead of incorporating an exogenous tag sequence, a complementary sequence derived from the two ends of the gene was added to each of the internal primers. Base pairs 3094 through 3402, corresponding to amino acids 1032 through 1134, were deleted from the *crbS* gene.

Deletion of VC2702 (*crbR*) was also performed using a similar approach, except that primers ED01 and ED02 amplified the upstream sequence, and primers ED03 and ED05 amplified the downstream sequence in SIO. Deletion of the REC domain from VC2702 in SIO was performed using primers ED80, ED81, ED82, and ED83, which deleted base pairs 12 through 342, corresponding to amino acids 4 through 114, from the gene. The complementary sequence that allows for self-annealing of the fragment was internal to the gene. For both the Δ*crbR* and *crbR*ΔREC constructs, the PCR product was amplified with Q5 polymerase (NEB) and directly ligated into pHC001B following digestion with *BamH*I and *Spe*I. The ligations were then transformed into DH5-αλ*pir* cells. The *acs* deletion was constructed using the Gibson method (NEB) for direct ligation into pHC001B. Incorporation of the insert into pHC001B was verified by digestion or by colony PCR using the PT64 and PT66 primers. Conjugation into *V*. *cholerae* was performed as described above.

#### Two-component system point mutants

Point mutations were constructed in the *V*. *cholerae* SIO chromosome in residues hypothesized to be critical for putative phosphotransfer activity in CrbS using SOE PCR. We identified His-798 in the HisKA domain and Asp-1081 in the REC domain based upon alignments constructed in the SMART web resource [37] and BLASTP [38]. We engineered mutations into overlapping SOE primers to convert the His-798 residue to Ala (GCG). The construct was generated by amplifying the upstream sequence with primers AEP234 and AEP235 and amplifying the downstream sequence with primers AEP236 and AEP237. Primers AEP235 and AEP236 overlap one another and carry the targeted mutation. To mutate His-798 to Gln (CAA), primers AEP234, AEP238, AEP239, and AEP237 were used. The Asp-1081 residue was converted to Ala (GCG) with primers AEP240, AEP241, AEP242, and AEP243. As described previously, the PCR product was amplified with Q5 polymerase (NEB) and directly ligated into pHC001B following digestion with *Sac*I and *Spe*I. The ligations were then transformed into DH5-αλ*pir* cells, and plasmids carrying constructs verified by sequencing were electroporated into MFD*pir* and conjugated into *V*. *cholerae* as described earlier. Integration of the H798A or H798Q mutations into the *V*. *cholerae crbS* gene was verified using primers AEP234 and AEP237 to amplify the surrounding region, and primer AEP233 for sequencing. Integration of the D1081A mutation into *crbS* was verified by amplifying surrounding sequence with primers AEP240 and AEP243, followed by sequencing with primer ED47.

#### Construction of pBBRlux transcriptional fusion plasmid to the *acs* promoter and introduction into *V*. *cholerae* strains

To construct the transcriptional fusion to the *luxCDABE* operon, a 660-bp region of the *acs* promoter was amplified using the PT47 and PT49 primers, digested with *BamH*I and *Spe*I, and ligated into pBBRlux [27], generating pPT002. The pPT002 plasmid was transformed into S17-1λ*pir E*. *coli*, and conjugated into *V*. *cholerae*. Transformants were selected on ampicillin and chloramphenicol plates, since the SIO strain is naturally ampicillin resistant and carries the *bla* gene (data not shown).

#### Luminescence assays

Bacterial strains carrying the empty pBBRlux plasmid or the pPT002 plasmid containing the *acs* promoter were grown in LBM with 5 μg/mL chloramphenicol for 14 to 15 hours. The cultures were then diluted 1:500 into 12 mL LBM-chloramphenicol (5 μg/mL) in 50 mL conical bioreactor tubes (Corning) and incubated with shaking at 250 rpm at 37°C. The OD_600nm_ was measured in a spectrophotometer (Jenway 6320D), and luminescence was detected using a GloMax 20/20 luminometer (Promega). Statistical significance was examined using the Mann-Whitney test in Prism 7 (GraphPad).

#### Role of *crbS*, *crbR*, *and acsA* in acetate metabolism utilizing M63 minimal media

To determine whether mutations in *crbS*, *crbR*, or *acsA* have an effect on acetate metabolism in non-O1/non-O139 strains of *V*. *cholerae*, strains carrying these mutations, as well as deletions in REC domains of *crbS* and *crbR*, were grown on M63 media (VWR) with 15mM supplemental sodium acetate (Sigma). Single colonies were grown on fresh LB plates overnight at 37°C, inoculated into LBM media, and grown overnight with shaking at 200 rpm at 37°C. The bacteria were spun down at 8000 *g* for 3 minutes, the supernatant was removed, and bacteria were resuspended in M63 media with 15mM sodium acetate to a final OD of 0.010 in 125 mL Erlenmeyer flasks. The bacteria were grown with shaking at 200 rpm at 37°C, and the optical density was measured at 600 nm on a spectrophotometer.

#### Infection of *Drosophila* with *Vibrio* and *Pseudomonas*

Bacterial strains from fresh plates were inoculated into LBM broth and incubated overnight with shaking at 37°C. Cultures were then diluted 1:10 in fresh LBM broth. Cellulose acetate plugs were cut into approximately 1.25 cm thick circular slices, and individual plug slices were placed at the bottom of fly vials (Genesee Scientific). Two milliliters of inoculated broth were added to each acetate plug slice. Ten male OregonR flies (stock originally from Michele Markstein, University of Massachusetts Amherst) between 4 and 10 days old were added to each vial. Each bacterial strain was tested in triplicate in each assay, alongside flies fed LBM broth alone as controls. At least three biological replicates of each assay were performed. Fly survival was monitored twice daily for at least five days, and statistical significance of survival curves was assessed using the log-rank test in Prism 7 (GraphPad).

## Results and discussion

### CrbS/R homologs regulate *acsA* expression in *Pseudomonas* and non-O1/non-O139 *V*. *cholerae*

**Fig 1:**
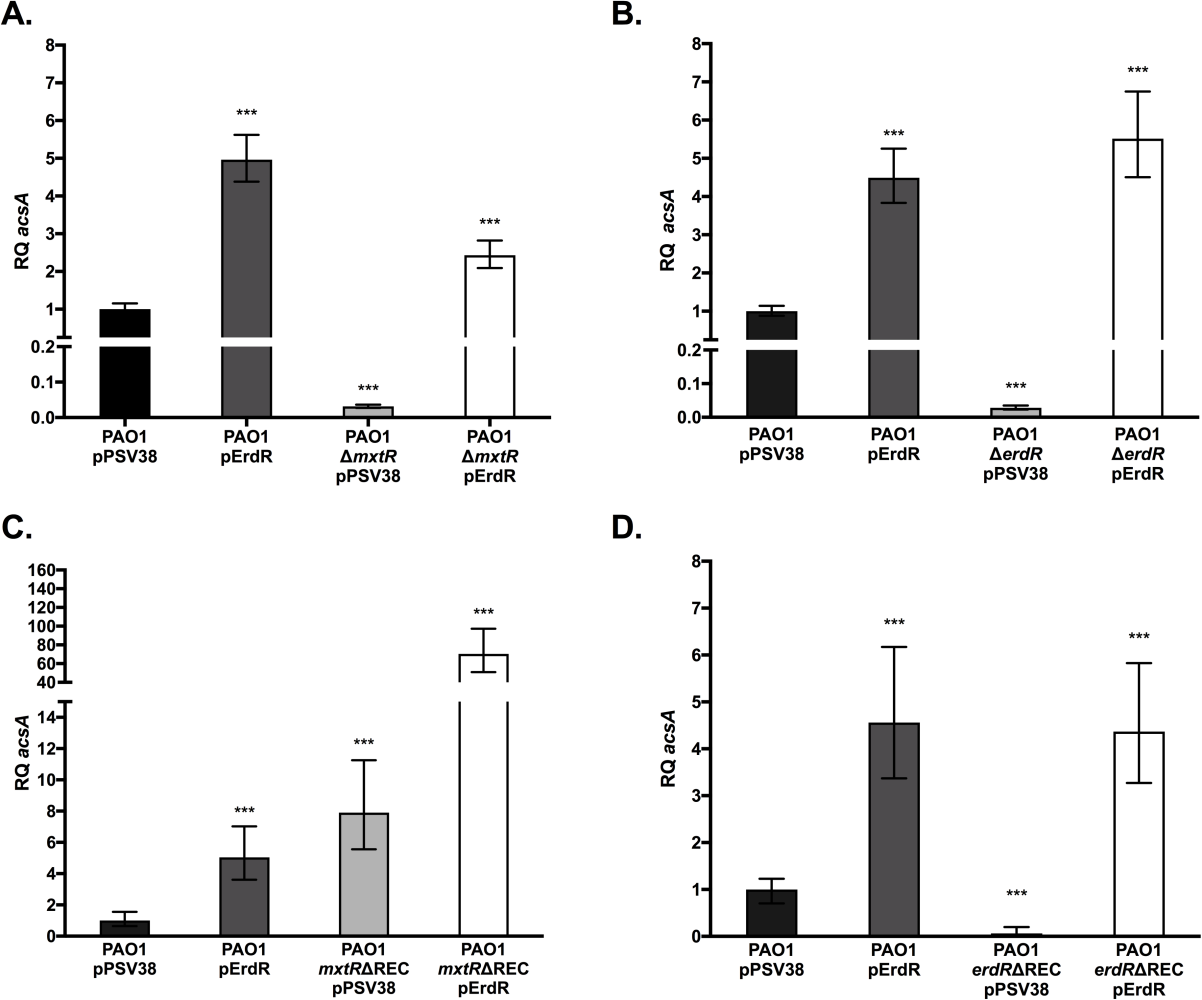
CrbS/R homologs control expression of *acsA* in *Pseudomonas aeruginosa*. Quantitative real-time PCR was used to measure *acsA* transcript abundance in an *mxtR* sensor kinase mutant (A), *erdR* response regulator mutant (B), and REC domain mutants of either *mxtR* (C) or *erdR* (D) in *Pseudomonas aeruginosa*. *acsA* transcript levels were measured relative to the *clpX* housekeeping protease transcript levels. Strains were transformed with either an empty vector plasmid pPSV38, or a pPSV38-*erdR* (pErdR) expression vector, as indicated. Statistical significance was determined by comparing results of each mutant strain to the wild-type strain. (*) denotes a P-value less than 0.05, (**) denotes a P-value less than 0.01, and (***) denotes a P-value less than 0.001.

During late exponential phase and early stationary phase, CrbS/R upregulates *acsA* in toxigenic O139 strains of *V*. *cholerae* [7]. We hypothesized that the homologs of this TCS in *P*. *aeruginosa* and *P*. *entomophila*, as well as those in non-O1/non-O139 *V*. *cholerae* strains, would similarly regulate *acsA* expression. To test this hypothesis, qRT-PCR was utilized to measure the transcript abundance of *acsA* in the sensor kinase and response regulator mutants of *P*. *aeruginosa* and *P*. *entomophila* compared to that of the wild-type strains. Each of the strains was transformed with an empty vector (pPSV38). Relative abundance of *acsA* transcript levels was normalized to the transcript levels of the housekeeping gene *clpX* for both organisms. qRT-PCR analysis of the *P*. *aeruginosa* Δ*mxtR* sensor kinase mutant showed a 31-fold decrease in *acsA* expression compared to that of the wild-type strain (Fig 1A). In the Δ*erdR* response regulator mutant, *acsA* expression was decreased 16-fold relative to that of the wild-type strain (Fig 1B). To confirm that the reduction in *acsA* expression was linked to interruptions in this pathway, both deletion mutant strains were transformed with a plasmid that overexpressed the response regulator of this TCS (pPSV38-*erdR*). In both complementation strains, wild-type levels of *acsA* expression were restored (Fig 1A and 1B). Interestingly, overexpression of *erdR* in the wild-type *P*. *aeruginosa* strain resulted in significantly higher expression of *acsA* (Fig 1).

**Fig 2:**
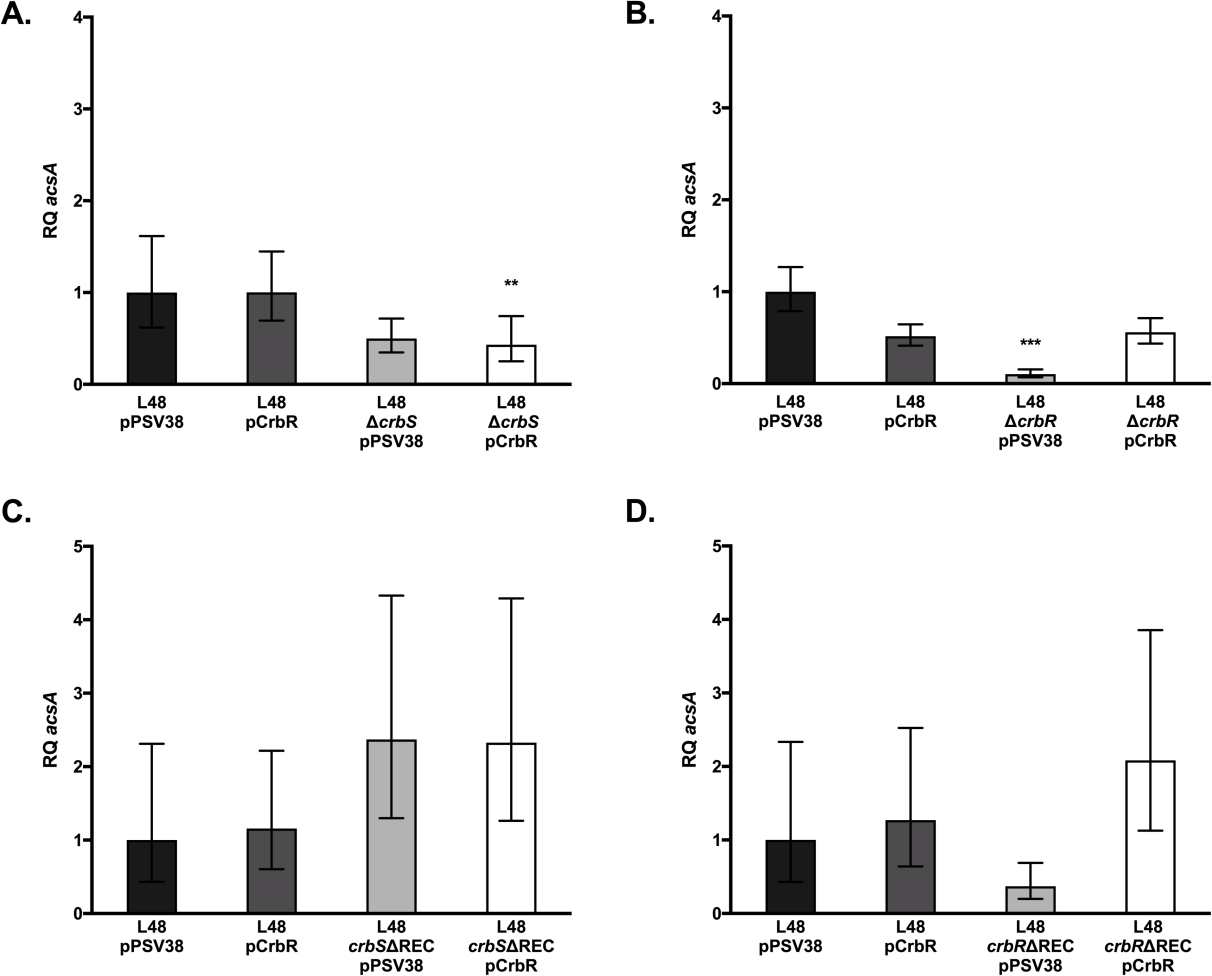
CrbS/R homologs control expression of *acsA* in *Pseudomonas entomophila*. Quantitative real-time polymerase chain reaction was used to measure *acsA* transcript abundance in a *crbS* sensor kinase mutant (A), *crbR* response regulator mutant (B), and REC domain mutants of either *crbS* (C) or *crbR* (D) in *Pseudomonas entomophila*. *acsA* transcript levels were measured relative to the *clpX* housekeeping protease transcript levels. Strains were transformed with either an empty vector plasmid, pPSV38, or a pPSV38-*crbR* (pCrbR) expression vector. Statistical significance was determined by comparing results for each mutant strain to those of the wild-type strain. (*) denotes a P-value less than 0.05, (**) denotes a P-value less than 0.01, and (***) denotes a P-value less than 0.001.

In *P*. *entomophila*, qRT-PCR analysis showed a 2-fold decrease in *acsA* expression of the Δ*crbS* sensor kinase mutant compared to that of the wild-type strain transformed with an empty vector (pPSV38) (Fig 2A), although this difference did not reach statistical significance. Deletion of the *crbR* response regulator had a greater effect on *acsA* expression, decreasing levels of *acsA* 4-fold compared to those of the wild-type strain (Fig 2B). In contrast to the *P*. *aeruginosa* results, when *P*. *entomophila crbS* and *crbR* mutants were transformed with plasmids that overexpress CrbR, wild-type levels of *acsA* expression were restored in the *crbR* mutant, but were not in the *crbS* mutant. Furthermore, overexpression of *crbR* in the wild-type *P*. *entomophila* strain did not result in a significant increase in *acs* expression levels. These results suggest that overexpression of *crbR* in *P*. *entomophila* does not lead to a large increase in active CrbR protein. Alternatively, the configuration of the signaling pathway may differ between the two species.

**Fig 3:**
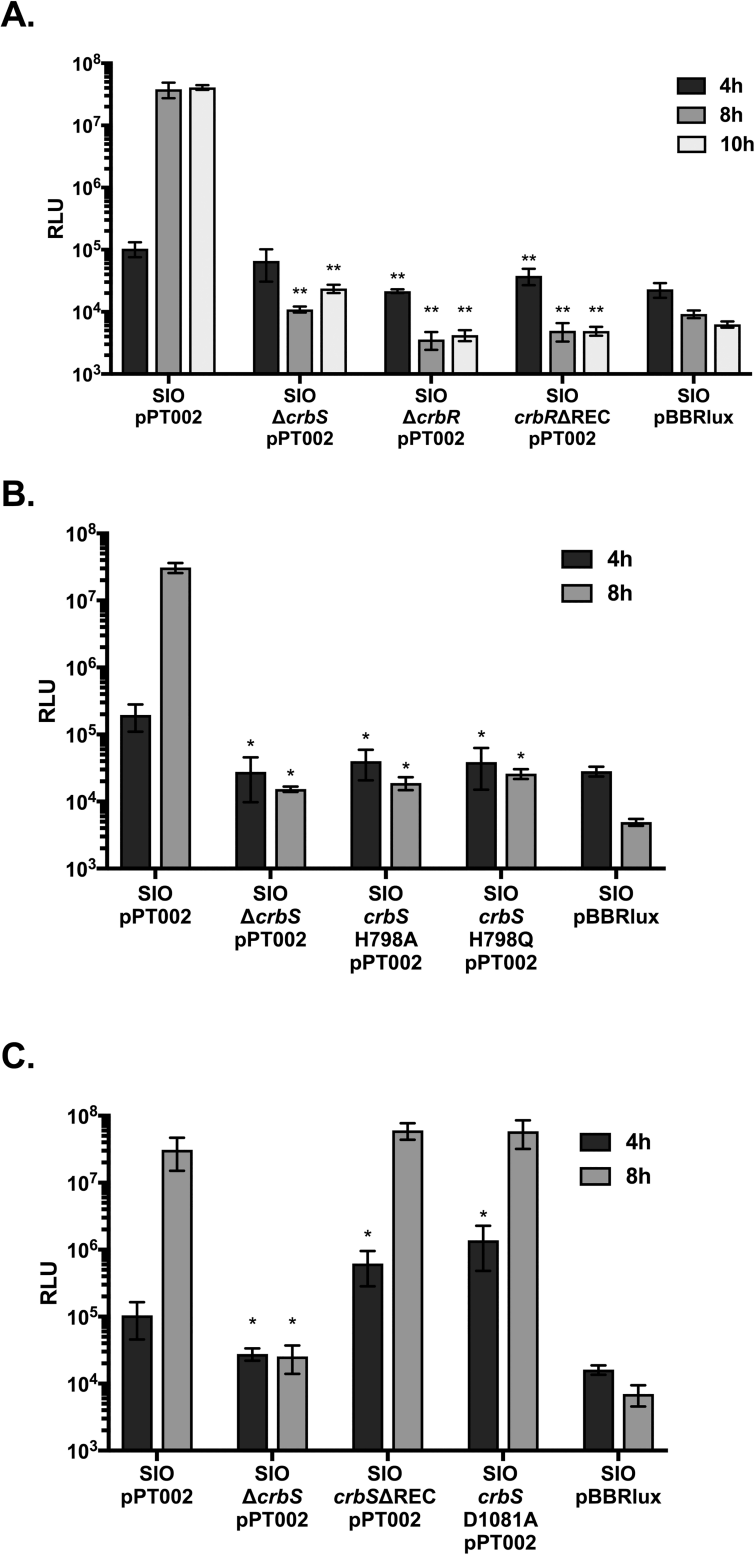
CrbS/R controls expression of *acsA* in a non-O1/non-O139 strain of *V*. *cholerae*. Expression of the *luxCDABE* operon driven by the *V*. *cholerae* SIO *acsA* promoter in plasmid pPT002 was measured after 4, 8, and 10 hours of growth, and normalized to OD_600nm_. The pBBRlux plasmid carries no promoter sequence. Luminescence was measured in *V*. *cholerae* strains carrying in-frame deletions of *crbS*, *crbR*, or the receiver domain of *crbR* (A); in *V*. *cholerae* strains with mutations in the putative conserved histidine within the HisKA domain (H798A and H798Q) (B); and in *V*. *cholerae* strains carrying either an in-frame deletion of the *crbS* receiver domain or a mutation within the putative conserved aspartate residue in the *crbS* receiver domain (D1081A) (C). Results from two biological replicates, each performed in duplicate or triplicate, are shown. Statistical significance was determined by comparing results from each mutant strain to those of the wild-type strain at that time point using the Mann-Whitney test. (*) denotes a P-value less than 0.05, (**) denotes a P-value less than 0.01, and (***) denotes a P-value less than 0.001.

To examine expression of *acsA* in the non-O1/non-O139 strain of *V*. *cholerae*, SIO, a 660-bp fragment of the *acs* promoter region was cloned into the pBBRlux plasmid and introduced into *V*. *cholerae* by conjugation. Transcription of the *acs* promoter was monitored by measuring luminescence relative to optical density. Deletion of either the *crbS* or *crbR* genes abrogated *acs* transcription after 8 and 10 hours of incubation (Fig 3A), echoing results seen previously in toxigenic strains of *V*. *cholerae* [7].

### Phosphotransfer domains modulate signaling through this TCS

In TCSs, signaling is mediated by phosphotransfer between a conserved histidine in the histidine kinase A (HisKA) domain and a conserved aspartate in the REC domain. The HisKA and REC domains can be arranged in a number of different configurations. In the simplest system, HK carries a single HisKA domain and the response regulator carries a single REC domain. More complex systems can involve two or more phosphotransfer events between a series of HisKA and REC domains on three or more proteins [39]. The CrbS sensor kinase carries both a HisKA domain and a REC domain, and CrbR carries a single REC domain. This suggests that the CrbS could function as a hybrid HK, facilitating phosphotransfer between the His in the CrbS HisKA domain, the Asp in the CrbS REC domain, a second His on an unknown HPt domain–containing protein, and a final Asp in the CrbR REC domain. To test the hypothesis that the conserved His residue within the HisKA domain is required for signaling, we engineered mutations in His-798 in the chromosomal copy of the *V*. *cholerae* SIO *crbS* gene by substituting this residue for either Ala and Gln. Both mutations reduced *acsA* expression significantly, and prevented induction of the acetate switch (Fig 3B). While this result is consistent with the hypothesis that His-798 is required for phosphotransfer, it is also possible that these mutations affect expression or folding of the CrbS protein to alter CrbS activity nonspecifically.

Next, to test the hypothesis that REC domains in both CrbS and CrbR are required for signaling in this system, we deleted these domains in the CrbS and CrbR homologs in *V*. *cholerae* SIO, *P*. *aeruginosa*, and *P*. *entomophila*. In *P*. *aeruginosa*, deletion of the REC domain of ErdR (*erdR*ΔREC) resulted in a 15-fold decrease in the relative abundance of *acsA* transcript (Fig 1D). Expression of *acsA* in an *erdR*ΔREC mutant was complemented when the strain was transformed with a plasmid expressing wild-type ErdR (pErdR) (Fig 1D). In *P*. *entomophila*, deletion of the REC domain of the *crbR* response regulator (*crbR*ΔREC) tended to reduce levels of *acsA* transcription, but the effect of the mutation did not reach statistical significance (Fig 2D). To determine whether overexpression of *crbR* could increase *acsA* expression in the *crbR*ΔREC background, we transformed the strain with a plasmid expressing wild-type CrbR (pCrbR). Expression of *acsA* was elevated in this strain, although the significance was not high (P = 0.022) (Fig 2D). This suggests that *crbR* may be capable of inducing low levels of *acs* expression even in the absence of the REC domain in *P*. *entomophila*. In contrast, deletion of the REC domain of CrbR completely abrogated *acsA* transcription in *V*. *cholerae* SIO (Fig 3A), indicating that the signaling function of the CrbR REC domain is conserved in both *V*. *cholerae* and *Pseudomonas*.

Unexpectedly, the deletions in the REC domains of the CrbS hybrid HK homologs did not reduce expression of *acsA* in any of the strains examined, and in some cases, expression of *acsA* was increased in these strains. In *P*. *aeruginosa*, deletion of the MxtR REC domain increased expression of *acsA* slightly (Fig 1C), and overexpression of ErdR in the *mxtR*ΔREC background resulted in a drastic increase in expression of *acsA* (Fig 1C). This suggests that both the CrbS REC domain and the amount of CrbR protein negatively regulate signaling through the pathway in *P*. *aeruginosa*. Similarly, deletion of the CrbS REC domain in *P*. *entomophila* resulted in a trend towards increased *acsA* expression, but this trend did not reach statistical significance (Fig 2C). Overexpression of CrbR protein in this background did not significantly raise *acsA* expression levels (Fig 2C). In *V*. *cholerae* SIO, removal of the CrbS REC domain did not affect *acsA* transcription positively or negatively after the acetate switch was flipped (Fig 3C). However, deletion of the REC domain increased expression of *acsA* at 4 hours of growth, prior to induction of the switch (Fig 3C). Because deletion of an entire domain of a protein may interfere with function in unexpected ways, we further engineered a specific point mutation in a conserved Asp residue, Asp-1081, in the REC domain of CrbS in *V*. *cholerae*, and monitored *acsA* transcription. The CrbS protein carrying this mutation acted similarly to that carrying the REC domain deletion, and increased expression of *acsA* prior to the switch. After the switch, *acsA* levels were indistinguishable from the wild-type (Fig 3C). These results provide additional evidence that phosphotransfer via the REC domain of CrbS is not a mandatory step in this signaling pathway. Altogether, these results support a model in which phosphotransfer occurs directly between CrbS His in the HisKA domain and the CrbR Asp in its REC domain, bypassing the CrbS Asp residue altogether. Alternatively, the CrbSΔREC kinase may cross-talk with another REC domain to continue the three-step pathway via an intermediary Hpt domain–containing protein in the absence of its REC domain. Observations of increased *acsA* expression in the CrbS REC domain deletion background support the hypothesis that the REC domain acts as a negative regulator of signaling, perhaps by functioning as a “phosphate sink”. Experiments to test this hypothesis are underway.

### Regulation of additional genes that may contribute to acetate metabolism by CrbS/R homologs

**Fig 4:**
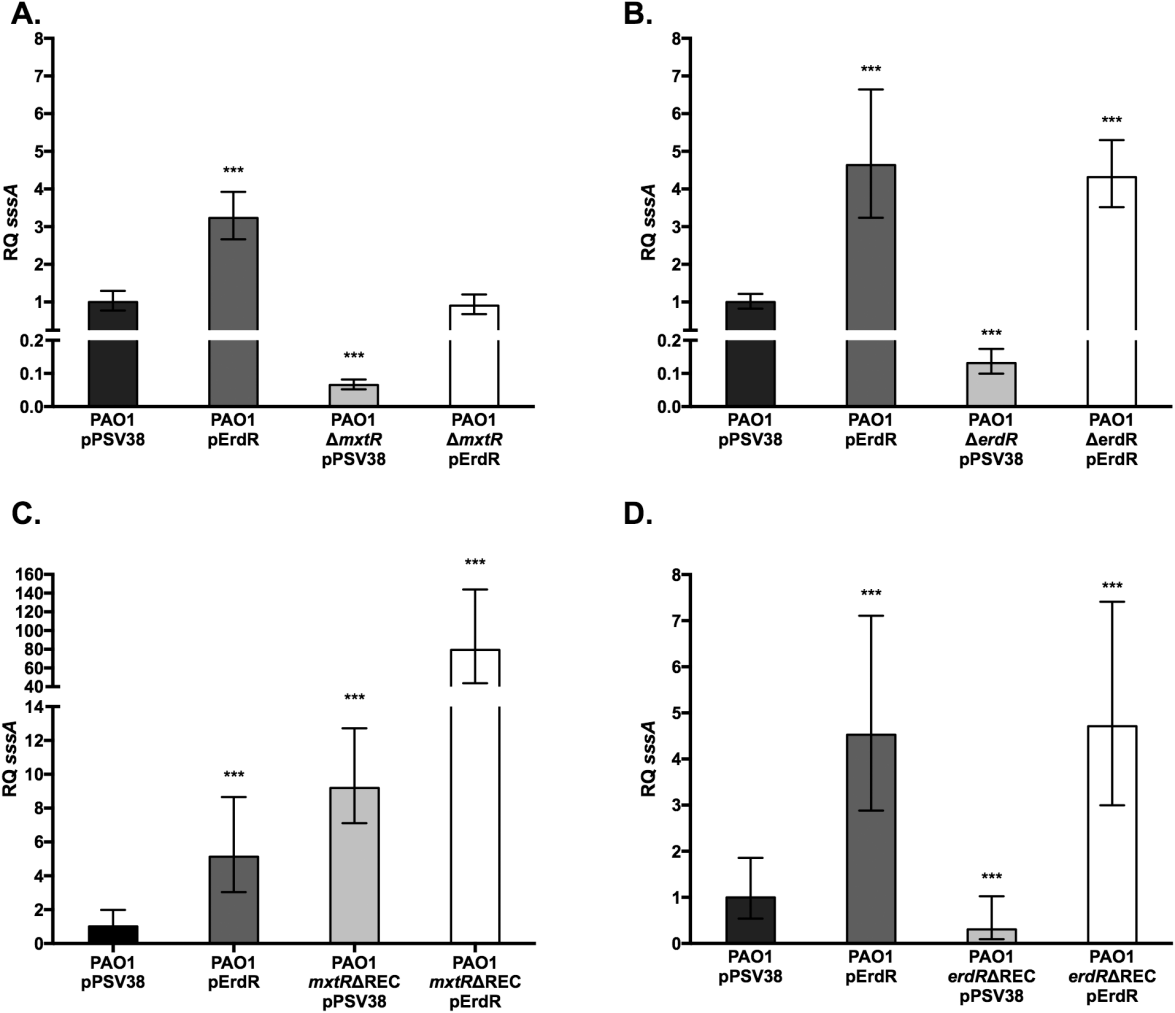
CrbS/R homologs control expression of an *sssA* homolog in *Pseudomonas aeruginosa*. Quantitative real-time polymerase chain reaction was used to measure the transcript abundance of an *sssA* homolog in an *mxtR* sensor kinase mutant (A), *erdR* response regulator mutant (B), and receiver domain mutants of either *mxtR* (C) or *erdR* (D) in *Pseudomonas aeruginosa*. *sssA* transcript levels were measured relative to the *clpX* housekeeping protease transcript levels. Strains were transformed with either an empty vector plasmid pPSV38 or a pPSV38-*erdR* (pErdR) expression vector. Statistical significance was determined by comparing results of each mutant strain to the wild-type strain. (*) denotes a P-value less than 0.05, (**) denotes a P-value less than 0.01, and (***) denotes a P-value less than 0.001.

RNAseq analysis of gene expression in *V*. *cholerae* has indicated that, in addition to *acsA*, expression of a putative acetate permease, *sssA*, was also highly regulated by the CrbS/R TCS [7]. Our previous results suggest that the homologous *P*. *aeruginosa* TCS regulates genes that are important for acetate metabolism. A BLASTN search of the *P*. *aeruginosa* genome identified PA3234 as the putative acetate permease that is most similar to *sssA* (44% identical). In *P*. *aeruginosa*, deletion of *mxtR* resulted in a 16-fold decrease in PA3234 expression relative to wild-type (Fig 4A) and deletion of *erdR* resulted in 8-fold decrease in PA3234 expression (Fig 4B). In a *P*. *aeruginosa erdR* REC domain mutant, PA3234 expression was decreased 3-fold (Fig 4D). Transformation of the Δ*mxtR*, Δ*erdR*, and *erdR*ΔREC mutants with a plasmid expressing wild-type ErdR complemented PA3234 expression (Fig 4A, 4B and 4D). Deletion of the *mxtR* REC domain increased PA3234 expression, and overexpression of ErdR in this background drastically raised PA3234 expression levels (Fig 4C), echoing observations of *acsA* transcription in these mutants (Fig 1). These results demonstrate that multiple members of the MxtR/ErdR regulon are conserved between *P*. *aeruginosa* and *V*. *cholerae*, and are subject to the same regulatory controls on the pathway. However, expression of a putative acetate transporter in *P*. *entomophila* did not exhibit similar patterns (data not shown). Given the difficulty of accurately finding homologous transporters in different organisms, it is possible that this gene is not the actual homolog.

### CrbS/R homologs are required for growth on acetate as sole carbon source

**Fig 5:**
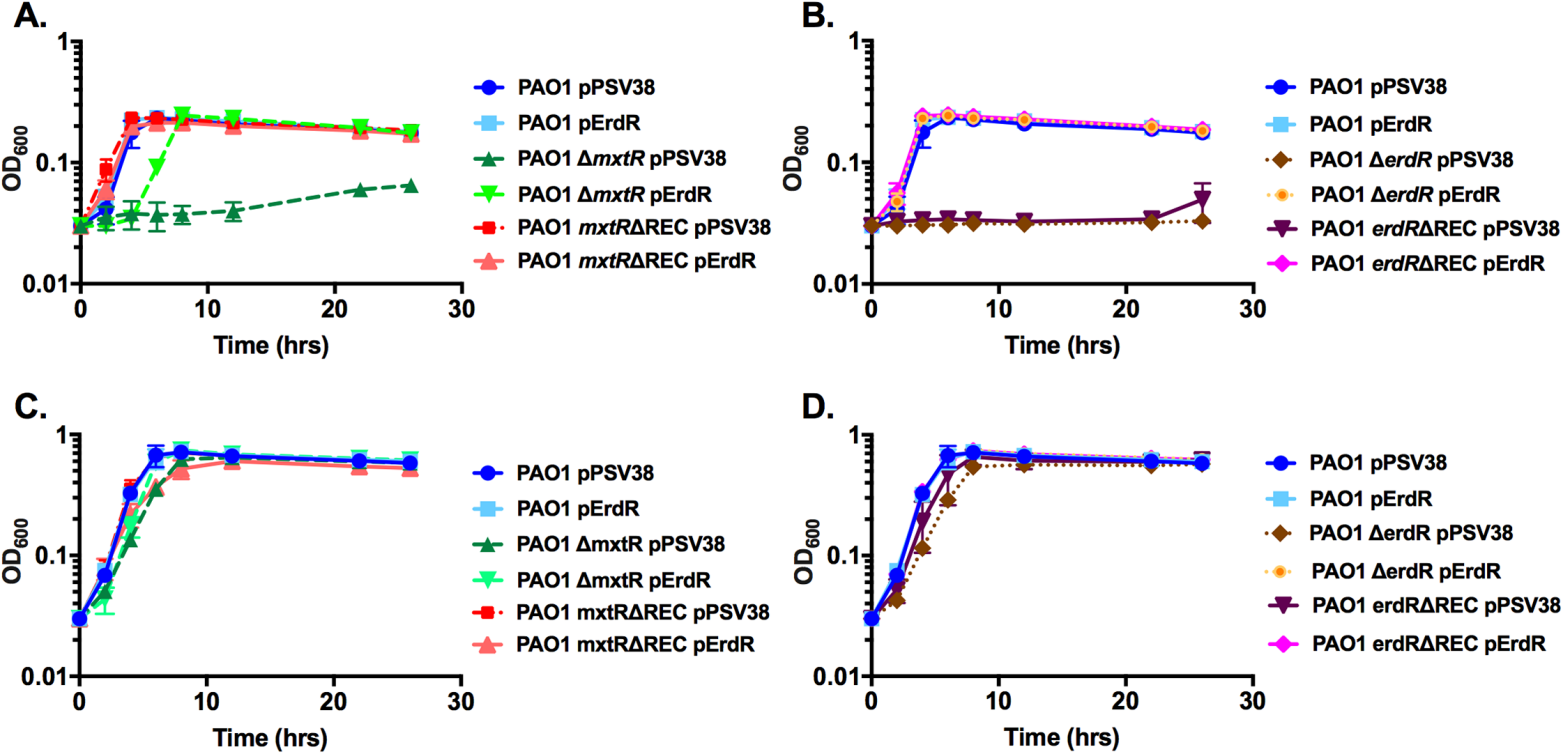
Homologs of the CrbS/R system are important for *Pseudomonas aeruginosa* growth on media with acetate as the sole carbon source. *P*. *aeruginosa* strains were inoculated to a starting OD_600nm_ of 0.03 in M63 minimal media supplemented with 5mM acetate as the sole carbon source. Growth was observed over a 28-hour period, during which cell density was recorded at the indicated time points by measuring optical density at 600 nm (A–B). Growth assays compared an *erdR* deletion mutant and an *erdR* receiver domain mutant strain to wild-type *P*. *aeruginosa* (A), and compared an *mxtR* deletion mutant and an *mxtR* receiver domain mutant strain to wild-type *P*. *aeruginosa* (B). *P*. *aeruginosa* strains were inoculated to a starting OD_600nm_ of 0.03 in M63 minimal media supplemented with 5mM acetate and 5mM glucose as carbon sources (C–D). Growth was observed over a 28-hour period, and cell density was measured at an optical density of 600 nm at the indicated time points. Growth assays comparing an *erdR* deletion mutant and an *erdR* receiver domain mutant strain to wild-type *P*. *aeruginosa* (C). Growth assays comparing an a*mxtR* deletion mutant and an *mxtR* receiver domain mutant strain to wild-type *P*. *aeruginosa* (D). Strains were transformed with either an empty vector plasmid pPSV38 or a pErdR expression vector, as indicated.

**Fig 6:**
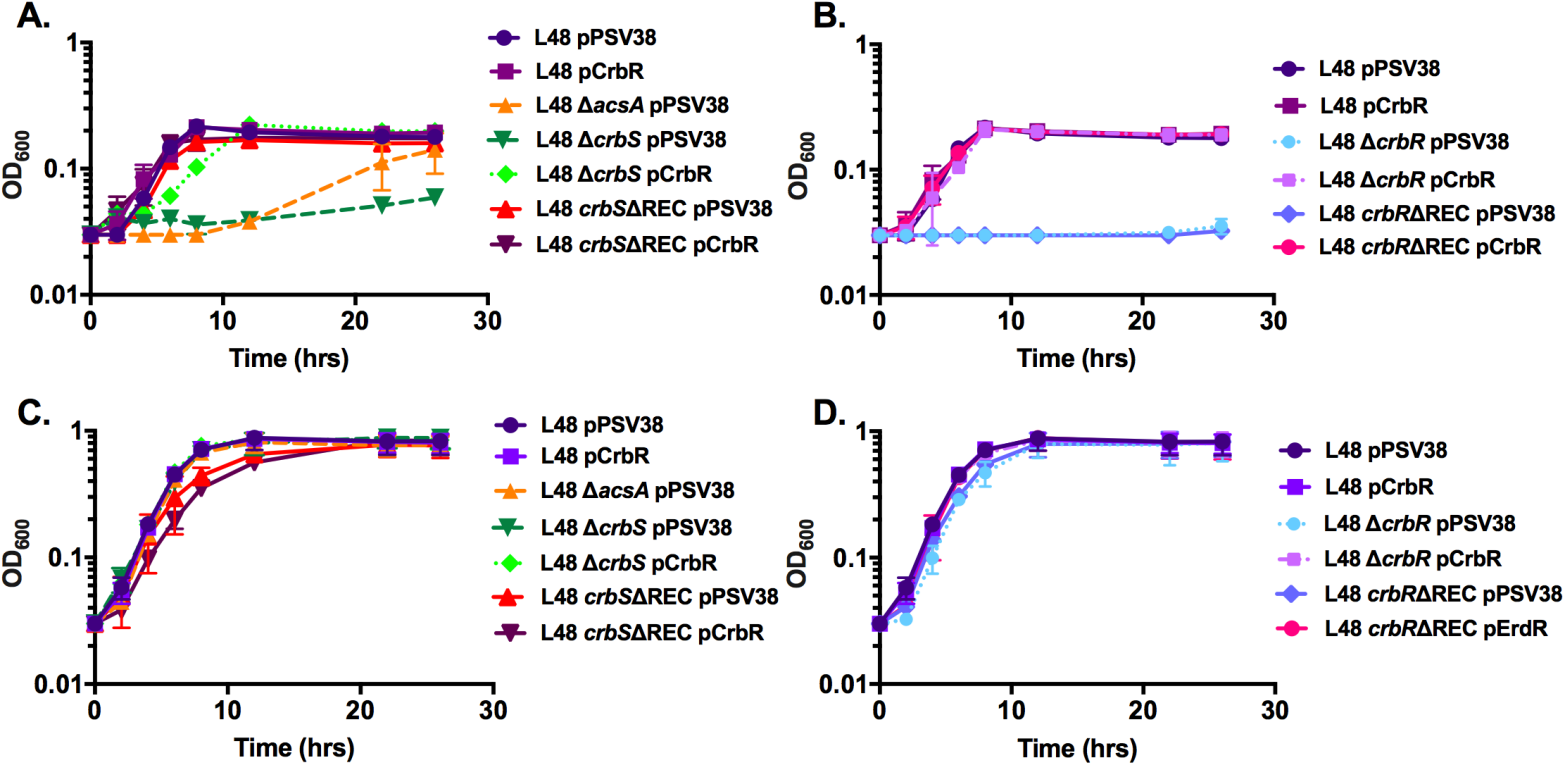
Homologs of the CrbS/R system are important for *Pseudomonas entomophila* growth on media with acetate as the sole carbon source. *Pseudomonas entomophila* strains were inoculated to a starting OD_600nm_ of 0.03 in M63 minimal media supplemented with 5mM acetate as the sole carbon source. Growth was observed over a 28-hour period, during which cell density was measured at an optical density of 600 nm at the indicated time points (A, B). Growth assays comparing a *crbS* deletion mutant, a *crbS* receiver domain mutant strain, and an *acsA* mutant strain to wild-type *P*. *entomophila* (A). Growth assays comparing a *crbR* deletion mutant and a *crbR* receiver domain mutant strain to wild-type *P*. *entomophila* (B). *P*. *entomophila* strains were inoculated to a starting OD_600nm_ of 0.03 in M63 minimal media supplemented with 5mM acetate and 5mM glucose as carbon sources (C, D). Growth was observed over a 28-hour period, and cell density was measured at an OD_600nm_ at the indicated time points. Growth assays compared a *crbS* deletion mutant strain, a *crbS* receiver domain mutant strain, and an *acsA* mutant strain to wild-type *P*. *entomophila* (C). Growth assays comparing a *crbR* deletion mutant and a *crbR* receiver domain mutant strain to wild-type *P*. *aeruginosa* (D). Strains were transformed with either an empty vector plasmid pPSV38 or a pCrbR expression vector, as indicated.

If the *P*. *aeruginosa* and *P*. *entomophila crbS*/*R* TCS plays a critical role in the regulation of genes involved in acetate metabolism, then we would expect that growth of Δ*acsA*, Δ*crbS*, Δ*mxtR*, Δ*erdR*, and Δ*crbR* mutants would be impaired in minimal media with acetate as the sole carbon source. Compared to wild-type *P*. *aeruginosa* and wild-type *P*. *entomophila*, strains with deletions of either the sensor kinases or the response regulators exhibited significantly reduced growth when grown in M63 media containing 5mM acetate as the sole carbon source (Figs 5A, 5B, 6A and 6B). Interestingly, *P*. *entomophila* strains containing a deletion of *acsA* initially exhibited a slow growth phenotype (Fig 6A). After approximately 16 hours, growth of the *acsA* mutant strain increased unexpectedly. We reasoned that this increase could be attributed to selection of suppressor mutations that rescue the repressed growth of the *acsA* deletion strain. To address this possibility, cultures of wild-type *P*. *entomophila* and the *acsA* mutant were inoculated into M63 media supplemented with 5mM acetate (M63/A) or M63 with both 5mM acetate and 5mM glucose (M63/AG). After 8, 24, 36, and 48 hours of growth, samples were plated onto agar with M63/A or M63/AG. After 8 hours, the *acsA* mutant was incapable of growing on M63/A agar (S1 Fig). However, when plated 24, 36, and 48 hours post-inoculation, small colonies of the *acsA* mutant were observed on M63/A (S2, S3 and S4 Figs). These colonies are likely suppressor mutants. This would explain the growth at the later time points in the growth assay as well as the larger error bars in the growth measurements. As expected, the *acsA* mutant grew on the M63/AG plates at all time points.

The PAO1 *erdR*ΔREC and L48 *crbR*ΔREC mutants, which carry deletions in the REC domains of the response regulator, also grew much more slowly in M63 media containing acetate as the sole carbon source than they did in media that contained both acetate and glucose (Figs 5B and 6B). When the Δ*crbS*, Δ*mxtR*, Δ*erdR*, Δ*crbR*, and *erdR*ΔREC mutants were transformed with a plasmid expressing ErdR or CrbR, growth in M63 media with acetate as the sole carbon source was restored to wild-type levels (Figs 5A, 5B, 6A and 6B). Interestingly, deletion of the REC domain of CrbS and MxtR did not significantly affect growth rates on acetate compared to those of the wild-type parental strains. Similar results were observed when these strains were transformed with one of the following plasmids: pPSV38 (empty vector), pPSV38-*erdR*, or pPV38-*crbR* (Figs 5A and 6A). If the reduced growth rates of the Δ*crbS*, Δ*erdR*, Δ*crbR*, and *erdR*ΔREC mutants are due to defects in the metabolism of acetate, then supplementing the M63 minimal media containing acetate with a second carbon source should restore normal growth to these mutants. When the Δ*crbS*, Δ*mxtR*, Δ*erdR*, Δ*crbR*, *crbS*ΔREC, *mxtR*ΔREC, and *erdR*ΔREC mutants were grown in M63 minimal media containing 5mM acetate and a second carbon source (5mM glucose), their growth rate did not vary significantly from the wild-type *P*. *aeruginosa* or wild-type *P*. *entomophila* strains (Figs 5C, 5D, 6C and 6D).

**Fig 7:**
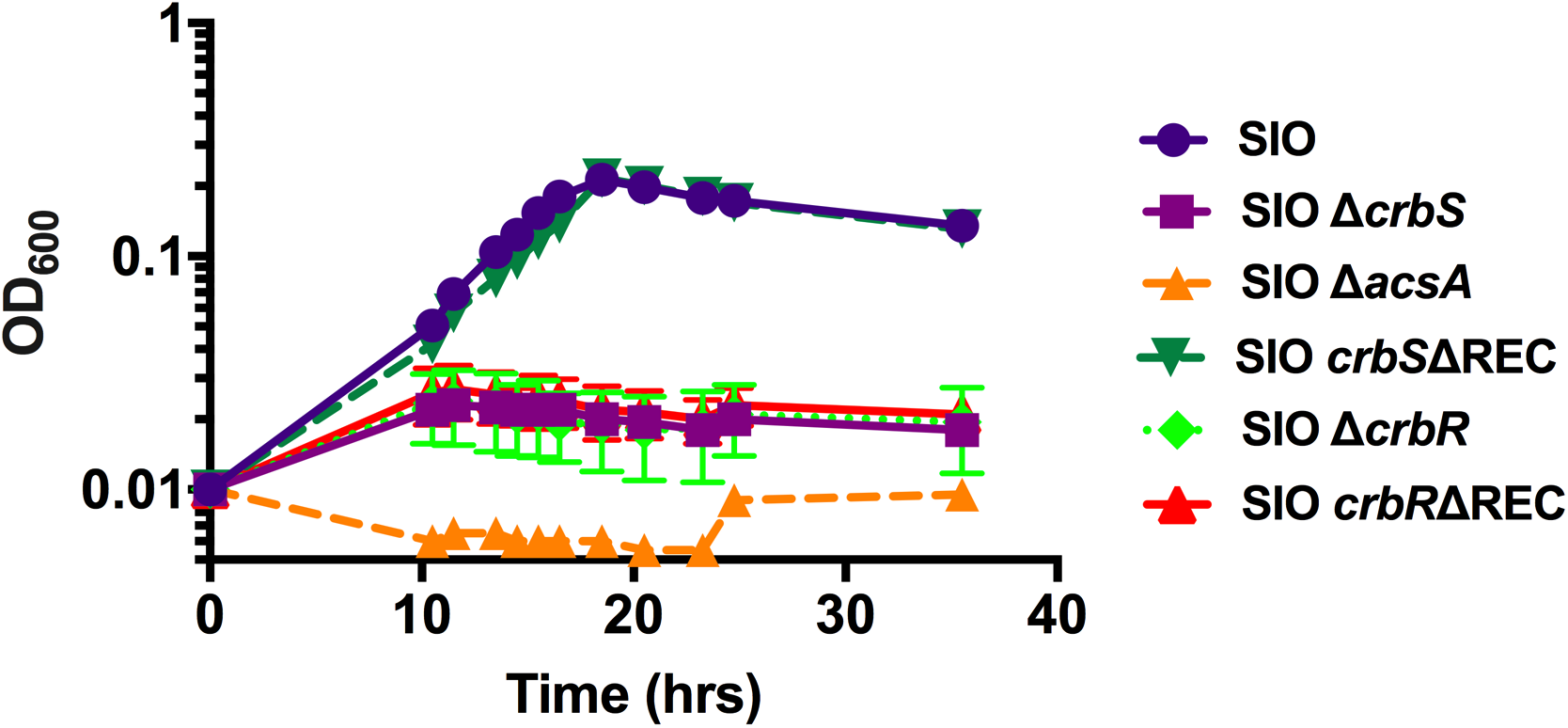
The CrbS/R system is important for *Vibrio cholerae* growth on media with acetate as the sole carbon source. *Vibrio cholerae* strains were inoculated to a starting OD_600nm_ of 0.005 in M63 minimal media supplemented with 15mM acetate. Growth was observed over a 36-hour period, and cell density was measured at an OD_600nm_ at the indicated time points.

In order to test whether mutations in *crbS* and *crbR* similarly affected the ability of *V*. *cholerae* to grow on acetate, we inoculated *V*. *cholerae* SIO wildtype, as well as *V*. *cholerae* SIO carrying deletions in *crbS*, *crbR*, or *acsA* into M63 media supplemented with 15 mM acetate as a sole carbon source. In this media, SIO wildtype reached a maximal OD of ~0.2 after 18 hours and SIO Δ*acsA* was unable to grow (Fig 7). Strains with deletions in *crbS*, *crbR*, or the REC domain of *crbR* displayed distinct growth impairments in this media. However, deletion of the *crbS* receiver domain did not affect growth under these conditions (Fig 7). These results are consistent with the conclusion that *crbS* and *crbR* are required for full expression of genes involved in acetate uptake, including *acs*. The *crbR* receiver domain is required for *crbR* function and signaling, while the *crbS* receiver domain does not play a role in activating utilization of acetate.

### CrbS/R homologs do not regulate hemolysin or AprA protease in *Pseudomonas*

Hemolysin and AprA protease production markedly affect virulence of *P*. *aeruginosa* and *P*. *entomophila* in *Drosophila* infection models [5,40]. The *Pseudomonas* CrbS/R homologs may regulate other virulence factors, such as hemolysin and AprA, that contribute to lethality in *Drosophila*. Expression of hemolysin and AprA protease were assayed in mutants of the *crbR* and *crbS* homologs. As a control, these virulence factors were also assayed in a strain carrying a deletion in *gacA*, a response regulator that positively regulates both hemolysin and AprA expression [5,41,42]. Wild-type *P*. *aeruginosa* and Δ*gacA*, Δ*mxtR*, and Δ*erdR* mutants were grown on blood agar or 5% milk agar to assay for hemolysin and AprA protease activity, respectively. While deletion of the *gacA* response regulator resulted in significantly decreased hemolysin and protease activity, no significant effect on hemolysin or protease production was observed in the Δ*mxtR* or Δ*erdR* strains (S5 Fig). Similar results were seen with *crbS/R* deletion mutants in *P*. *entomophila* (S6 Fig). These observations demonstrate that CrbS/R is not broadly involved in regulating virulence in *Pseudomonas* species, and instead may be involved with regulating central metabolic pathways. Testing this hypothesis will require further description of the CrbS/R regulon via RNASeq or other global regulatory methods in each of these species under varying environmental conditions.

### Effects of CrbS/R homologues on virulence of *P*. *aeruginosa*, *P*. *entomophila* and non-O1/non-O139 *V*. *cholerae* in a *Drosophila melanogaster* model of infection

**Fig 8:**
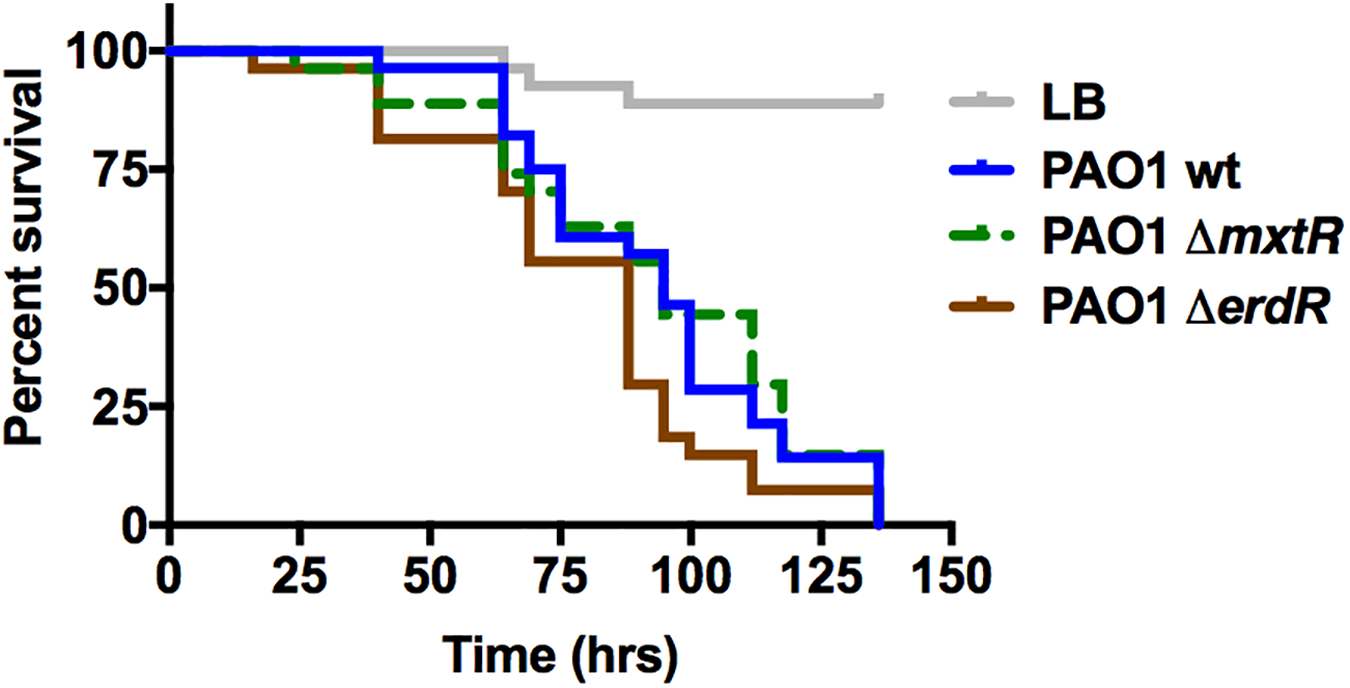
The CrbS/R homologs of *Pseudomonas aeruginosa* do not significantly contribute to virulence towards *Drosophila*. Survival of flies fed bacterial strains in Luria Bertani (Miller) broth was monitored for 136 hours in triplicate vials containing 10 flies each. Statistical significance of survival differences associated with each individual mutant strain relative to the wild-type strain was assessed using the log-rank test. None of the mutants displayed virulence phenotypes significantly different from that of the wild-type strain (P<0.05).

**Fig 9:**
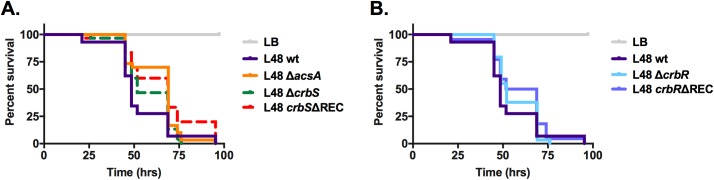
The CrbS/R homologs of *Pseudomonas entomophila* do not play a dominant role in determining virulence towards *Drosophila*. Survival of flies fed bacterial strains in Luria Bertani (Miller) broth was monitored for 125 hours in triplicate vials containing 10 flies each. Statistical significance of differences in fly survival associated with each mutant strain relative to the wild-type strain was assessed using the log-rank test. Some strains of *Pseudomonas entomophila* exhibited slight differences in fly survival; in this assay, the *crbS*ΔREC, *acsA*, and *crbR* mutants were associated with significantly different survival rates than was the wild-type strain (P = 0.0076, P = 0.0034, and P = 0.047, respectively) (A, B). However, none of the mutant strains differed significantly from the wild-type strain in all three biological replicates of the assay (S2 Table).

Wild-type *P*. *aeruginosa* and *P*. *entomophila* are known entomopathogens that can cause death in a *D*. *melanogaster* model of infection [1,5,6]. The CrbS/R TCS regulates virulence in toxigenic O139 strains of *V*. *cholerae* [7]. To determine whether the CrbS/R homologs regulate virulence in non-O1/non-O139 nontoxigenic *V*. *cholerae*, as well as *P*. *aeruginosa* or *P*. *entomophila*, fly survival assays were performed [43]. Flies ingested either LBM media, wild-type *P*. *aeruginosa*, *P*. *entomophila*, non-O1/non-O139 *V*. *cholerae* strain SIO, or mutants containing deletions in the genes encoding the CrbS and CrbR homologues. Virtually all flies feeding on uninoculated LB media survived. Flies feeding on LBM media containing wild-type *P*. *aeruginosa* or *P*. *entomophila* died within 150 hours (Figs 8 and 9). Deletion of *mxtR* or *erdR* in *P*. *aeruginosa* did not drastically reduce virulence (Fig 8), but log-rank analysis shows a significant difference in fly survival between *P*. *aeruginosa* Δ*mxtR* and wild-type *P*. *aeruginosa* in two of six assays (P = 0.0221 and P = 0.0135) (S2 Table). In just one of six assays, deletion of *erdR* significantly slowed fly survival relative to the wild-type strain (P = 0.0165) (S2 Table). Similarly, deletion of the Δ*crbS* and Δ*crbR* genes in *P*. *entomophila* did not drastically alter fly susceptibility to infection (Fig 9), although significance was reached in one of three assays (P = 0.0409 for *P*. *entomophila* Δ*crbS* and P = 0.0477 for *P*. *entomophila* Δ*crbR*) (S2 Table). To determine whether *acsA* plays a role in virulence, this deletion in *P*. *entomophila* was also tested in the fly survival assay, and again virulence reduction reached significance in one of three assays (P = 0.0034) (Fig 9; S2 Table). To further confirm these results, strains carrying mutations in the REC domains of CrbS and CrbR were also tested for their effects on fly survival. Deletion of the CrbR REC domain did not abrogate virulence in any of the three assays, but deletion of the CrbS REC domain did have a significant effect in two of three assays (P = 0.0076 and P = 0.008) (Fig 8B; S2 Table). These results indicate that this TCS does not play a dominant role in regulating the pathogenicity of either *P*. *aeruginosa* or *P*. *entomophila* towards *Drosophila*. However, small effects on virulence observed in a minority of assays suggest the possibility that this system could modulate virulence in a minor way.

**Fig 10:**
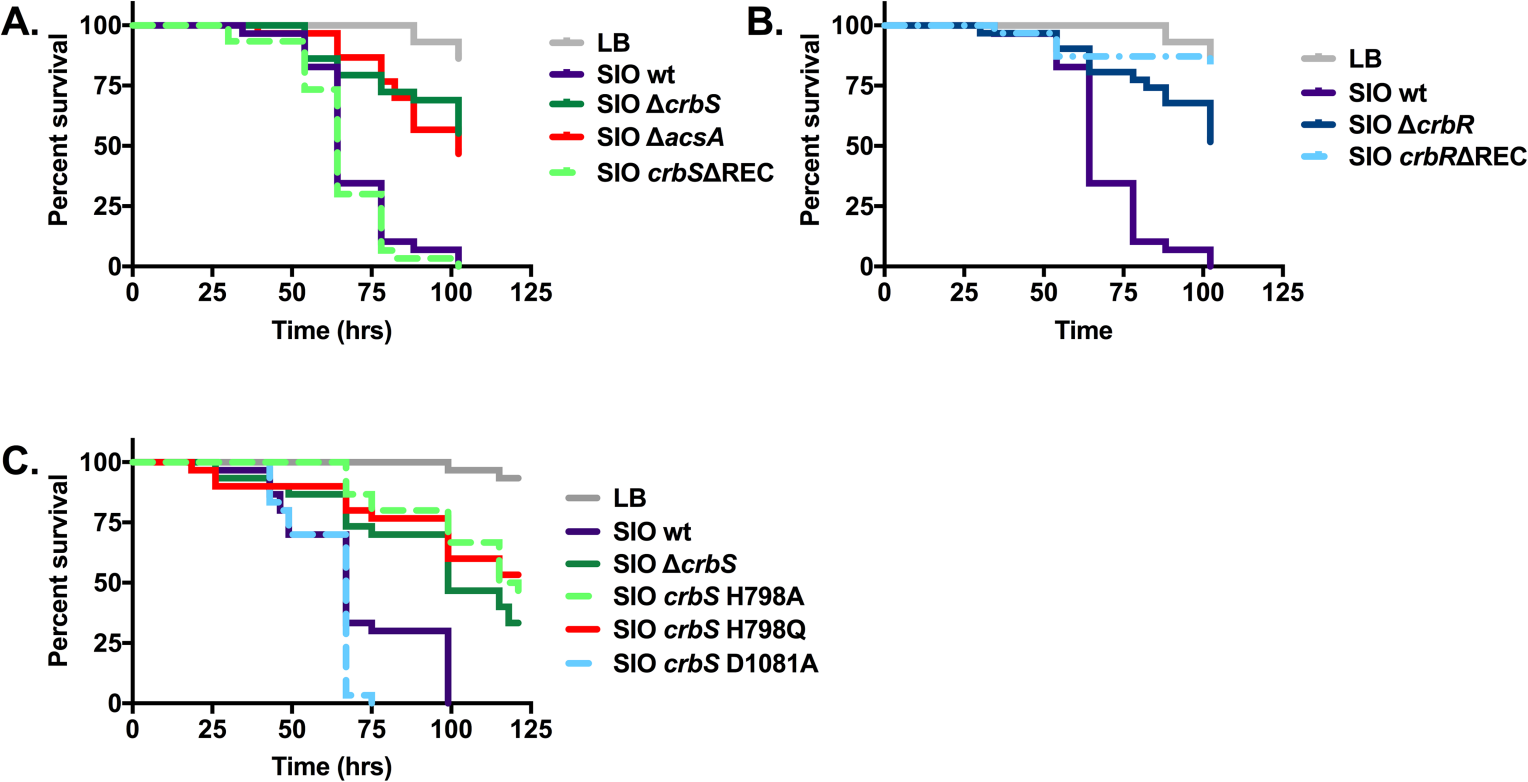
Mortality of *Drosophila* infected with *V*. *cholerae* strain SIO is dependent upon CrbS/R homologs. Survival of flies fed bacterial strains in LB broth was monitored for 110 to 125 hours in triplicate vials containing 10 flies each. Statistical significance of differences in fly survival associated with each mutant strain relative to that associated with the wild-type strain was assessed using the log-rank test. Flies infected with the *crbS*ΔREC mutant succumbed more quickly than did flies infected with the wild-type strain in this assay (P = 0.0028) (A), but this result was not reproduced in three additional assays (S2 Table). Survival of flies infected with the *crbS*, *crbR*, and *crbR*ΔREC mutants was significantly greater than that of flies infected with the wild-type strain (P<0.0001) (B). These results were reproduced in three additional independent experiments (S2 Table). Mutation of *crbS* residues important in the phosphorelay pathway demonstrate that mutation of CrbS His-798 to Ala or Gln reduces lethality of *V*. *cholerae* SIO (P<0.0001) in three independent assays (S2 Table), of which one representative example is shown (C). Mutation of CrbS Asp-1081 to Ala does not reduce lethality, but instead trends towards increasing virulence of *V*. *cholerae* SIO (P = 0.0354) in three independent assays (S2 Table).

In order to investigate the role of CrbS/R-dependent regulation of *acs* in a non-O1/non-O139 strain of *V*. *cholerae*, mutations of *acs*, *crbS*, and *crbR* in *V*. *cholerae* SIO were tested for their effects on virulence towards flies. Deletions of each of these genes resulted in a significant reduction of virulence in each of four assays (P<0.0001) (Fig 10; S2 Table). As expected, deletion of the CrbR REC domain in strain SIO also reduced virulence significantly (P<0.0001). Interestingly, deletion of the CrbS REC domain did not reduce virulence compared to the wild-type strain in three of four assays (P>0.05) (S2 Table). However, in one of four assays this deletion did result in significantly faster virulence (P = 0.0028) (S2 Table). These results suggest that CrbS-mediated virulence towards *Drosophila* is conserved in environmental strains of *V*. *cholerae*.

## Conclusions

In this work, we demonstrate that a novel regulator of central metabolic pathways is conserved in bacterial genera capable of colonizing and infecting a wide variety of host species. We provide evidence that the CrbS/R system functions as a signaling pathway to control expression of *acsA*, the gene that encodes acetyl-CoA synthetase, in non-O1/non-O139 *V*. *cholerae* strains, the human opportunistic pathogen *P*. *aeruginosa*, and the entomopathogen *P*. *entomophila*. This pathway is required for growth of these strains on acetate minimal media, suggesting that the physiological function of the pathway is also conserved. CrbS/R regulates virulence towards *Drosophila* in non-O1/non-O139 strains of *V*. *cholerae*, but does not appear to play a primary role in *Pseudomonas* virulence in this model. We also provide evidence that the CrbS REC domain is dispensable for signaling, and may function as a negative regulator of the pathway.

Acetyl-CoA synthetase is universally conserved among organisms from bacteria to humans, and serves as one of the primary mechanisms through which acetate is converted to acetyl-CoA, a metabolite at the intersection of central metabolic pathways involved in energy harvesting, fatty acid metabolism, and carbon catabolism [44]. Acs expression and activity is controlled by multiple layers of regulatory mechanisms that are conserved to varying degrees, suggesting that universal constraints on flux through this pathway must be balanced with species-specific needs for regulation. For example, control of Acs enzymatic activity by reversible lysine acetylation appears to be conserved across the domains of life [45–48]. In bacteria, transcription of *acs* responds both to widely conserved global regulators and to pathways that are narrowly distributed in small phylogenetic groupings. cAMP-CRP regulates *acs* in *E*. *coli* [44], but in *Vibrio fischeri acs* is controlled, at least in part, by the *Vibrio-*specific AinS quorum sensing mechanism [49]. We and others have demonstrated that CrbS and/or CrbR contribute to *acs* regulation in *V*. *cholerae*, *V*. *vulnificus*, *Pseudomonas*, and *Shewanella* [7,19–21], but the distribution of the CrbS/R genes suggests that they may function in other Gram-negative bacteria as well. However, the nature of the information delivered to the cell as a result of CrbS/R signaling is unknown. The structure of the CrbS protein, with a domain similar to a sodium-solute symporter at its N-terminus, strongly suggests that CrbS/R-dependent *acs* expression is linked to the sensing and/or transport of a specific molecule. Discovering this signal could help determine whether CrbS-carrying bacteria activate *acs* transcription in response to unique physiological circumstances. Alternatively, CrbS/R may respond to the same environmental cues as other pathways that regulate *acs*. Essentially, we do not yet know whether CrbS/R effectively supplants these other known regulatory pathways in some bacteria, or instead provides an additional layer of regulatory control in response to a novel signal.

As a first step towards interrogating this signaling pathway in more detail, the REC domains of CrbS and CrbR were deleted in *P*. *aeruginosa*, *P*. *entomophila*, and *V*. *cholerae* SIO. We hypothesized that the REC domains in each of these proteins would be necessary for signaling, as they are in many other TCSs that involve hybrid HKs, including RscS in *V*. *fischeri*, LuxN in *V*. *cholerae*, VirA in *Agrobacterium tumefaciens*, and BvgS in *Bordetella* [50–53]. As expected, removal of the REC domain of the CrbR homologs disrupted the pathway and prevented activation of *acs* expression in all three strains. Surprisingly, deletion of the REC domain in CrbS homologs did not reduce signaling, and under some conditions, removal of the REC domain instead facilitated activation of *acs* and growth on acetate minimal media. Similar results have been observed in biochemical and genetic studies of other phosphorelays, suggesting an alternative function for the REC domain of hybrid HKs under some conditions. In *Yersinia*, mutation of the conserved Asp residue within the REC domain of the hybrid HK YsrS, which regulates expression of the type III secretion system in response to NaCl, resulted in higher expression of the target gene [54]. Biochemical and genetic analyses of other phosphorelay pathways, including those that control chemotaxis, have implicated REC domains as negative regulators of phosphotransfer. Evidence suggests that the REC domain can act as a “phosphate sink” [55–58] by competing with a second, productive branch of the pathway for phosphorylation of its conserved Asp. Upon transfer, the phosphate is hydrolyzed, and thus signaling is effectively halted. We hypothesize that the CrbS REC domain is acting to finely tune signaling through the pathway by hydrolyzing phosphate. Experiments to test this biochemically are underway.

Although CrbS/R regulates expression of *acs* in both *Pseudomonas* and *Vibrio*, this signaling system does not play a similar role in the pathogenesis of the two organisms towards *Drosophila*. *V*. *cholerae* regulation of short chain fatty acid levels in the fly alimentary canal is critical to its success as a pathogen in an oral model of infection [7], but an *acs* mutant of *P*. *entomophila* was not similarly defective in virulence (Fig 9). Furthermore, the CrbS/R system did not significantly or consistently contribute to fly mortality resulting from infection with either *Pseudomonas* strain. These results suggest that *Pseudomonas*-dependent killing of *Drosophila* proceeds via mechanisms that operate independently of CrbS/R and acetate metabolism. Furthermore, previous studies of *P*. *entomophila* and *P*. *aeruginosa* fly infection have not provided evidence for a critical role for central metabolic pathways in mediating infection. However, metabolic genes that contribute to virulence may have been excluded from consideration because mutations in these genes are likely to result in growth defects. One transposon mutagenesis screen of >7000 individual *P*. *entomophila* mutants in a *Drosophila* oral infection assay uncovered 23 loci that contributed to virulence without compromising growth [41]. Few of the identified mutations affected metabolic processes: a gene involved in biotin biosynthesis and a putative transporter of amino acids were identified, but none were directly related to acetate metabolism. Although metabolic processes clearly underlie pathogenesis in a myriad of ways, it is particularly challenging to investigate the roles of specific genes because of the need to disentangle the effect of a change in the metabolome from the contribution of the potential growth defect.

Virulence of *P*. *aeruginosa* towards *Drosophila* has been investigated intensively for more than a decade. However, most studies have employed a model in which bacteria are introduced to the *Drosophila* hemocoel by pricking, and as a result have uncovered genes that aid systemic infection rather than factors that contribute to survival and disease within the alimentary canal. The development of an oral model of *P*. *aeruginosa* infection has facilitated the identification of numerous genes and physiological processes that may assist the pathogen in colonizing the intestine and overcoming the intestinal immune response [59–63]. To our knowledge, no studies to date have revealed a role for acetate metabolism in the virulence phenotype observed in *Pseudomonas* infection of *Drosophila*. Furthermore, CrbS/R does not appear to function as a global regulator of virulence in *Pseudomonas*, as it does not control expression of hemolysin or protease, two factors that can contribute to virulence towards both flies and humans [40,64–67], and virulence factors needed for *Drosophila* infection are functional in its absence.

Although CrbS/R does not contribute to pathogenesis in this model, conservation of the CrbS/R-Acs pathway suggests that it plays an important role under certain physiological and environmental conditions. Nutrient acquisition and fatty acid metabolism are important in *P*. *aeruginosa* lung infections of cystic fibrosis patients [68]. Several genes involved in fatty acid metabolism and the tricarboxylic acid cycle have been shown to be significantly upregulated in high cell density *P*. *aeruginosa* infections in the lungs of cystic fibrosis patients, and *acsA* was more highly expressed in a sputum isolate of *P*. *aeruginosa* than it was in PAO1 grown on citrate [68]. The possibility that CrbS/R-dependent acetate metabolism plays a role during certain periods of *P*. *aeruginosa* lung infection awaits further investigation.

New functions for secreted bacterial metabolites in host physiology are continually being uncovered, and deciphering regulatory mechanisms that control levels of bacterially-derived short-chain fatty acids is crucial to understanding how microbes alter the balance between health and disease [8]. CrbS/R represents a widely-conserved mechanism through which acetate can be regulated by a variety of host-associated Gram-negative bacteria. However, the molecular mechanisms through which CrbS/R functions, the signaling information it provides, and its role in mediating other host–microbe interactions remains to be explored.

## Supporting information

S1 Fig Deletion of *acsA* results in the selection of suppressor mutants that can survive on media with acetate as the sole carbon source: 8-hour time point. Eight hours postinoculation, 100 μl of culture was spread plated on M63-acetate or M63-acetate/glucose agar supplemented with 1mM IPTG and 30 μg/mL gentamicin as indicated. The strains shown are wild-type *Pseudomonas entomophila* + pPSV38 on M63 agar with 5mM acetate (A), wild-type *Pseudomonas entomophila* + pPSV38 on M63 agar with 5mM acetate and 5mM glucose (B), *Pseudomonas entomophila* Δ*acsA* + pPSV38 on M63 agar with 5mM acetate(C), and *Pseudomonas entomophila* Δ*acsA* + pPSV38 on M63 agar with 5mM acetate and 5mM glucose (D). Note the absence of growth when *Pseudomonas entomophila* Δ*acsA* is grown on M63 agar with 5mM acetate as the sole carbon source (C).
(TIF)

S2 Fig Deletion of *acsA* results in the selection of suppressor mutants that can survive on media with acetate as the sole carbon source: 24-hour time point. Twenty-four hours postinoculation, 10 μl of culture was quadrant streaked on M63-acetate or M63-acetate/glucose agar supplemented with 1mM IPTG and 30 μg/mL gentamicin as indicated. The strains shown are wild-type *Pseudomonas entomophila* + pPSV38 on M63 agar with 5mM acetate (A), wild-type *Pseudomonas entomophila* + pPSV38 on M63 agar with 5mM acetate and 5mM glucose (B), *Pseudomonas entomophila* Δ*acsA* + pPSV38 on M63 agar with 5mM acetate (C), and *Pseudomonas entomophila* Δ*acsA* + pPSV38 on M63 agar with 5mM acetate and 5mM glucose (D). Note the appearance of suppressor mutants when *Pseudomonas entomophila* Δ*acsA* is grown on M63 agar with 5mM acetate as the sole carbon source (C).
(TIF)

S3 Fig Deletion of *acsA* results in the selection of suppressor mutants that can survive on media with acetate as the sole carbon source: 36-hour time point. Thirty-six hours postinoculation, 10 μL of culture was quadrant streaked on M63-acetate or M63-acetate/glucose agar supplemented with 1mM IPTG and 30 μg/mL gentamicin, as indicated. The strains shown are wild-type *Pseudomonas entomophila* + pPSV38 on M63 agar with 5mM acetate (A), wild-type *Pseudomonas entomophila* + pPSV38 on M63 agar with 5mM acetate and 5mM glucose (B), *Pseudomonas entomophila* Δ*acsA* + pPSV38 on M63 agar with 5mM acetate (C), and *Pseudomonas entomophila* Δ*acsA* + pPSV38 on M63 agar with 5mM acetate and 5mM glucose (D). Note the appearance of suppressor mutants when *Pseudomonas entomophila* Δ*acsA* is grown on M63 agar with 5mM acetate as the sole carbon source (C).
(TIF)

S4 Fig Deletion of *acsA* results in the selection of suppressor mutants that can survive on media with acetate as the sole carbon source: 48-hour time point. Forty-eight hours postinoculation, 10 μL of culture was quadrant streaked on M63-acetate or M63-acetate/glucose agar supplemented with 1mM IPTG and 30 μg/mL gentamicin as indicated. The strains shown are wild-type *Pseudomonas entomophila* + pPSV38 on M63 agar with 5mM acetate (A), wild-type *Pseudomonas entomophila* + pPSV38 on M63 agar with 5mM acetate and 5mM glucose (B), *Pseudomonas entomophila* Δ*acsA* + pPSV38 on M63 agar with 5mM acetate (C), and *Pseudomonas entomophila* Δ*acsA* + pPSV38 on M63 agar with 5mM acetate and 5mM glucose (D). Note the appearance of suppressor mutants when *Pseudomonas entomophila* Δ*acsA* is grown on M63 agar with 5mM acetate as the sole carbon source (C).
(TIF)

S5 Fig The CrbS/R two-component system does not regulate hemolysin or secreted protease production in *Pseudomonas aeruginosa*. Hemolysin production (A) and AprA protease production (B) of wild-type *Pseudomonas aeruginosa* PAO1 and mutant strains containing deletions of either *crbR* or *crbS*, plated on blood agar or Luria Bertani (Miller) agar + 5% milk, respectively.
(TIF)

S6 Fig The CrbS/R two-component system does not regulate hemolysin or secreted protease production in *Pseudomonas entomophila*. Hemolysin production (A) and AprA protease production (B) of wild-type *Pseudomonas entomophila* L48 and mutants containing deletions of either *gacA*, *crbR*, or *crbS*. Strains were plated on blood agar for assessing hemolytic activity or modified low salt Luria Bertani (Miller) agar with 5% milk to assay for AprA protease activity.
(TIF)

S1 Table Primers used in this study. (DOCX)

S2 Table Statistical analyses of *Drosophila* survival curves following ingestion of *Pseudomonas entomophila*, *Pseudomonas aeruginosa*, or *Vibrio cholerae*. Statistical analyses were performed in GraphPad Prism via log-rank analysis. Significance was tested relative to survival of flies that had ingested wild-type bacterial strains. Shaded blocks indicate assays in which the survival curves of the flies differed significantly from those of flies that had ingested wild-type strains (P<0.05). Blocks with bold text indicate assays in which flies died significantly faster than did flies that had ingested the wild-type strains (P<0.05). Blocks without shading or bold text indicate assays in which flies died at a rate that differed insignificantly from flies that ingested wild-type strains (P>0.05). NT, not tested.
(DOCX)

## References

[1] Tzou P, De Gregorio E, Lemaitre B. How Drosophila combats microbial infection: a model to study innate immunity and host–pathogen interactions. Curr Opin Microbiol. 2002;5: 102–110. 11834378 10.1016/s1369-5274(02)00294-1

[2] Panayidou S, Ioannidou E, Apidianakis Y. Human pathogenic bacteria, fungi, and viruses in Drosophila. Virulence. 2014;5: 253–269. doi: 10.4161/viru.27524 24398387 10.4161/viru.27524 PMC3956501

[3] Fauvarque M-O. Small flies to tackle big questions: assaying complex bacterial virulence mechanisms using Drosophila melanogaster. Cell Microbiol. 2014;16: 824–833. doi: 10.1111/cmi.12292 24628939 10.1111/cmi.12292

[4] Vodovar N, Acosta C, Lemaitre B, Boccard F. Drosophila: a polyvalent model to decipher host–pathogen interactions. Trends Microbiol. 2004;12: 235–242. doi: 10.1016/j.tim.2004.03.007 15120143 10.1016/j.tim.2004.03.007

[5] Vallet-Gely I, Opota O, Boniface A, Novikov A, Lemaitre B. A secondary metabolite acting as a signalling molecule controls Pseudomonas entomophila virulence. Cell Microbiol. 2010;12: 1666–1679. doi: 10.1111/j.1462-5822.2010.01501.x 20597908 10.1111/j.1462-5822.2010.01501.x

[6] Apidianakis Y, Rahme LG. Drosophila melanogaster as a model host for studying Pseudomonas aeruginosa infection. Nat Protoc. 2009;4: 1285–1294. doi: 10.1038/nprot.2009.124 19680242 10.1038/nprot.2009.124

[7] Hang S, Purdy AE, Robins WP, Wang Z, Mandal M, Chang S, et al The Acetate Switch of an Intestinal Pathogen Disrupts Host Insulin Signaling and Lipid Metabolism. Cell Host Microbe. 2014;16: 592–604. doi: 10.1016/j.chom.2014.10.006 25525791 10.1016/j.chom.2014.10.006 PMC4272434

[8] Koh A, De Vadder F, Kovatcheva-Datchary P, Bäckhed F. From Dietary Fiber to Host Physiology: Short-Chain Fatty Acids as Key Bacterial Metabolites. Cell. 2016;165: 1332–1345. doi: 10.1016/j.cell.2016.05.041 27259147 10.1016/j.cell.2016.05.041

[9] Shin SC, Kim S-H, You H, Kim B, Kim AC, Lee K-A, et al Drosophila Microbiome Modulates Host Developmental and Metabolic Homeostasis via Insulin Signaling. Science. 2011;334: 670–674. doi: 10.1126/science.1212782 22053049 10.1126/science.1212782

[10] Vinolo MAR, Rodrigues HG, Nachbar RT, Curi R. Regulation of Inflammation by Short Chain Fatty Acids. Nutrients. 2011;3: 858–876. doi: 10.3390/nu3100858 22254083 10.3390/nu3100858 PMC3257741

[11] Brestoff JR, Artis D. Commensal bacteria at the interface of host metabolism and the immune system. Nat Immunol. 2013;14: 676–684. doi: 10.1038/ni.2640 23778795 10.1038/ni.2640 PMC4013146

[12] Purdy A, Rohwer F, Edwards R, Azam F, Bartlett DH. A Glimpse into the Expanded Genome Content of Vibrio cholerae through Identification of Genes Present in Environmental Strains. J Bacteriol. 2005;187: 2992–3001. doi: 10.1128/JB.187.9.2992-3001.2005 15838025 10.1128/JB.187.9.2992-3001.2005 PMC1082809

[13] Vodovar N, Vinals M, Liehl P, Basset A, Degrouard J, Spellman P, et al Drosophila host defense after oral infection by an entomopathogenic Pseudomonas species. Proc Natl Acad Sci U S A. 2005;102: 11414–11419. doi: 10.1073/pnas.0502240102 16061818 10.1073/pnas.0502240102 PMC1183552

[14] Korycinski M, Albrecht R, Ursinus A, Hartmann MD, Coles M, Martin J, et al STAC–a new domain associated with transmembrane solute transport and two-component signal transduction systems. J Mol Biol. 2015; 10.1016/j.jmb.2015.08.017 26321252

[15] Zaoui C, Overhage J, Löns D, Zimmermann A, Müsken M, Bielecki P, et al An orphan sensor kinase controls quinolone signal production via MexT in Pseudomonas aeruginosa. Mol Microbiol. 2012;83: 536–547. doi: 10.1111/j.1365-2958.2011.07947.x 22168309 10.1111/j.1365-2958.2011.07947.x

[16] Nishijyo T, Haas D, Itoh Y. The CbrA–CbrB two-component regulatory system controls the utilization of multiple carbon and nitrogen sources in Pseudomonas aeruginosa. Mol Microbiol. 2001;40: 917–931. 11401699 10.1046/j.1365-2958.2001.02435.x

[17] Yeung ATY, Bains M, Hancock REW. The Sensor Kinase CbrA Is a Global Regulator That Modulates Metabolism, Virulence, and Antibiotic Resistance in Pseudomonas aeruginosa. J Bacteriol. 2011;193: 918–931. doi: 10.1128/JB.00911-10 21169488 10.1128/JB.00911-10 PMC3028677

[18] Zhang X-X, Gauntlett JC, Oldenburg DG, Cook GM, Rainey PB. Role of the Transporter-Like Sensor Kinase CbrA in Histidine Uptake and Signal Transduction. J Bacteriol. 2015;197: 2867–2878. doi: 10.1128/JB.00361-15 26148710 10.1128/JB.00361-15 PMC4524029

[19] Kretzschmar U, Khodaverdi V, Adrian L. Transcriptional regulation of the acetyl-CoA synthetase gene acsA in Pseudomonas aeruginosa. Arch Microbiol. 2010;192: 685–690. doi: 10.1007/s00203-010-0593-5 20549193 10.1007/s00203-010-0593-5

[20] Kim MJ, Kim J, Lee HY, Noh HJ, Lee K-H, Park S-J. Role of AcsR in expression of the acetyl-CoA synthetase gene in Vibrio vulnificus. BMC Microbiol. 2015;15: 86 doi: 10.1186/s12866-015-0418-4 25887971 10.1186/s12866-015-0418-4 PMC4409781

[21] Deutschbauer A, Price MN, Wetmore KM, Shao W, Baumohl JK, Xu Z, et al Evidence-Based Annotation of Gene Function in Shewanella oneidensis MR-1 Using Genome-Wide Fitness Profiling across 121 Conditions. PLoS Genet. 2011;7: e1002385 doi: 10.1371/journal.pgen.1002385 22125499 10.1371/journal.pgen.1002385 PMC3219624

[22] Simon R, Priefer U, Pühler A. A Broad Host Range Mobilization System for In Vivo Genetic Engineering: Transposon Mutagenesis in Gram Negative Bacteria. Nat Biotechnol. 1983;1: 784–791.

[23] Miller VL, Mekalanos JJ. A novel suicide vector and its use in construction of insertion mutations: osmoregulation of outer membrane proteins and virulence determinants in Vibrio cholerae requires toxR. J Bacteriol. 1988;170: 2575–2583. 2836362 10.1128/jb.170.6.2575-2583.1988 PMC211174

[24] Merrell DS, Camilli A. Regulation of vibrio cholerae genes required for acid tolerance by a member of the “ToxR-like” family of transcriptional regulators. J Bacteriol. 2000;182: 5342–5350. 10986235 10.1128/jb.182.19.5342-5350.2000 PMC110975

[25] Ferrières L, Hémery G, Nham T, Guérout A-M, Mazel D, Beloin C, et al Silent Mischief: Bacteriophage Mu Insertions Contaminate Products of Escherichia coli Random Mutagenesis Performed Using Suicidal Transposon Delivery Plasmids Mobilized by Broad-Host-Range RP4 Conjugative Machinery. J Bacteriol. 2010;192: 6418–6427. doi: 10.1128/JB.00621-10 20935093 10.1128/JB.00621-10 PMC3008518

[26] Stover CK, Pham XQ, Erwin AL, Mizoguchi SD, Warrener P, Hickey MJ, et al Complete genome sequence of Pseudomonas aeruginosa PAO1, an opportunistic pathogen. Nature. 2000;406: 959–964. doi: 10.1038/35023079 10984043 10.1038/35023079

[27] Lenz DH, Mok KC, Lilley BN, Kulkarni RV, Wingreen NS, Bassler BL. The Small RNA Chaperone Hfq and Multiple Small RNAs Control Quorum Sensing in Vibrio harveyi and Vibrio cholerae. Cell. 2004;118: 69–82. doi: 10.1016/j.cell.2004.06.009 15242645 10.1016/j.cell.2004.06.009

[28] Borisova SA, Christman HD, Metcalf MEM, Zulkepli NA, Zhang JK, Donk WA van der, et al Genetic and Biochemical Characterization of a Pathway for the Degradation of 2-Aminoethylphosphonate in Sinorhizobium meliloti 1021. J Biol Chem. 2011;286: 22283–22290. doi: 10.1074/jbc.M111.237735 21543322 10.1074/jbc.M111.237735 PMC3121374

[29] Rietsch A, Vallet-Gely I, Dove SL, Mekalanos JJ. ExsE, a secreted regulator of type III secretion genes in Pseudomonas aeruginosa. Proc Natl Acad Sci. 2005;102: 8006–8011. doi: 10.1073/pnas.0503005102 15911752 10.1073/pnas.0503005102 PMC1142391

[30] Castang S, McManus HR, Turner KH, Dove SL. H-NS family members function coordinately in an opportunistic pathogen. Proc Natl Acad Sci. 2008;105: 18947–18952. doi: 10.1073/pnas.0808215105 19028873 10.1073/pnas.0808215105 PMC2596223

[31] Ferrieres L, Hemery G, Nham T, Guerout A-M, Mazel D, Beloin C, et al Silent Mischief: Bacteriophage Mu Insertions Contaminate Products of Escherichia coli Random Mutagenesis Performed Using Suicidal Transposon Delivery Plasmids Mobilized by Broad-Host-Range RP4 Conjugative Machinery. J Bacteriol. 2010;192: 6418–6427. doi: 10.1128/JB.00621-10 20935093 10.1128/JB.00621-10 PMC3008518

[32] Choi K-H, Schweizer HP. mini-Tn7 insertion in bacteria with single attTn7 sites: example Pseudomonas aeruginosa. Nat Protoc. 2006;1: 153–161. doi: 10.1038/nprot.2006.24 17406227 10.1038/nprot.2006.24

[33] Goldman SR, Sharp JS, Vvedenskaya IO, Livny J, Dove SL, Nickels BE. NanoRNAs prime transcription initiation in vivo. Mol Cell. 2011;42: 817–825. doi: 10.1016/j.molcel.2011.06.005 21700226 10.1016/j.molcel.2011.06.005 PMC3130991

[34] Wolfgang MC, Lee VT, Gilmore ME, Lory S. Coordinate regulation of bacterial virulence genes by a novel adenylate cyclase-dependent signaling pathway. Dev Cell. 2003;4: 253–263. 12586068 10.1016/s1534-5807(03)00019-4

[35] Livak KJ, Schmittgen TD. Analysis of Relative Gene Expression Data Using Real-Time Quantitative PCR and the 2−ΔΔCT Method. Methods. 2001;25: 402–408. doi: 10.1006/meth.2001.1262 11846609 10.1006/meth.2001.1262

[36] Watve SS, Chande AT, Rishishwar L, Mariño-Ramírez L, Jordan IK, Hammer BK. Whole-Genome Sequences of 26 Vibrio cholerae Isolates. Genome Announc. 2016;4: e01396–16. 10.1128/genomeA.01396-16 PMC5180380 28007852

[37] Letunic I, Doerks T, Bork P. SMART: recent updates, new developments and status in 2015. Nucleic Acids Res. 2015;43: D257–D260. doi: 10.1093/nar/gku949 25300481 10.1093/nar/gku949 PMC4384020

[38] Johnson M, Zaretskaya I, Raytselis Y, Merezhuk Y, McGinnis S, Madden TL. NCBI BLAST: a better web interface. Nucleic Acids Res. 2008;36: W5–W9. doi: 10.1093/nar/gkn201 18440982 10.1093/nar/gkn201 PMC2447716

[39] Capra EJ, Laub MT. Evolution of Two-Component Signal Transduction Systems. Annu Rev Microbiol. 2012;66: 325–347. doi: 10.1146/annurev-micro-092611-150039 22746333 10.1146/annurev-micro-092611-150039 PMC4097194

[40] Liehl P, Blight M, Vodovar N, Boccard F, Lemaitre B. Prevalence of Local Immune Response against Oral Infection in a Drosophila / Pseudomonas Infection Model. PLOS Pathog. 2006;2: e56 doi: 10.1371/journal.ppat.0020056 16789834 10.1371/journal.ppat.0020056 PMC1475658

[41] Vodovar N, Vallenet D, Cruveiller S, Rouy Z, Barbe V, Acosta C, et al Complete genome sequence of the entomopathogenic and metabolically versatile soil bacterium Pseudomonas entomophila. Nat Biotechnol. 2006;24: 673–679. doi: 10.1038/nbt1212 16699499 10.1038/nbt1212

[42] Ventre I, Goodman AL, Vallet-Gely I, Vasseur P, Soscia C, Molin S, et al Multiple sensors control reciprocal expression of Pseudomonas aeruginosa regulatory RNA and virulence genes. Proc Natl Acad Sci U S A. 2006;103: 171–176. doi: 10.1073/pnas.0507407103 16373506 10.1073/pnas.0507407103 PMC1324988

[43] Blow NS, Salomon RN, Garrity K, Reveillaud I, Kopin A, Jackson FR, et al Vibrio cholerae Infection of Drosophila melanogaster Mimics the Human Disease Cholera. PLoS Pathog. 2005;1: e8 doi: 10.1371/journal.ppat.0010008 16201020 10.1371/journal.ppat.0010008 PMC1238743

[44] Wolfe AJ. The acetate switch. Microbiol Mol Biol Rev MMBR. 2005;69: 12–50. doi: 10.1128/MMBR.69.1.12-50.2005 15755952 10.1128/MMBR.69.1.12-50.2005 PMC1082793

[45] Starai VJ, Celic I, Cole RN, Boeke JD, Escalante-Semerena JC. Sir2-Dependent Activation of Acetyl-CoA Synthetase by Deacetylation of Active Lysine. Science. 2002;298: 2390–2392. doi: 10.1126/science.1077650 12493915 10.1126/science.1077650

[46] Hentchel KL, Escalante-Semerena JC. Acylation of Biomolecules in Prokaryotes: a Widespread Strategy for the Control of Biological Function and Metabolic Stress. Microbiol Mol Biol Rev. 2015;79: 321–346. doi: 10.1128/MMBR.00020-15 26179745 10.1128/MMBR.00020-15 PMC4503791

[47] Hallows WC, Lee S, Denu JM. Sirtuins deacetylate and activate mammalian acetyl-CoA synthetases. Proc Natl Acad Sci. 2006;103: 10230–10235. doi: 10.1073/pnas.0604392103 16790548 10.1073/pnas.0604392103 PMC1480596

[48] Schwer B, Bunkenborg J, Verdin RO, Andersen JS, Verdin E. Reversible lysine acetylation controls the activity of the mitochondrial enzyme acetyl-CoA synthetase 2. Proc Natl Acad Sci. 2006;103: 10224–10229. doi: 10.1073/pnas.0603968103 16788062 10.1073/pnas.0603968103 PMC1502439

[49] Studer SV, Mandel MJ, Ruby EG. AinS quorum sensing regulates the Vibrio fischeri acetate switch. J Bacteriol. 2008;190: 5915–5923. doi: 10.1128/JB.00148-08 18487321 10.1128/JB.00148-08 PMC2519518

[50] Geszvain K, Visick KL. The Hybrid Sensor Kinase RscS Integrates Positive and Negative Signals To Modulate Biofilm Formation in Vibrio fischeri. J Bacteriol. 2008;190: 4437–4446. doi: 10.1128/JB.00055-08 18441062 10.1128/JB.00055-08 PMC2446786

[51] Freeman JA, Lilley BN, Bassler BL. A genetic analysis of the functions of LuxN: a two-component hybrid sensor kinase that regulates quorum sensing in Vibrio harveyi. Mol Microbiol. 2000;35: 139–149. 10632884 10.1046/j.1365-2958.2000.01684.x

[52] Wise AA, Fang F, Lin Y-H, He F, Lynn DG, Binns AN. The Receiver Domain of Hybrid Histidine Kinase VirA: an Enhancing Factor for vir Gene Expression in Agrobacterium tumefaciens. J Bacteriol. 2010;192: 1534–1542. doi: 10.1128/JB.01007-09 20081031 10.1128/JB.01007-09 PMC2832513

[53] Uhl MA, Miller JF. Autophosphorylation and phosphotransfer in the Bordetella pertussis BvgAS signal transduction cascade. Proc Natl Acad Sci. 1994;91: 1163–1167. 8302847 10.1073/pnas.91.3.1163 PMC521474

[54] Walker KA, Obrist MW, Mildiner-Earley S, Miller VL. Identification of YsrT and Evidence that YsrRST Constitute a Unique Phosphorelay System in Yersinia enterocolitica. J Bacteriol. 2010;192: 5887–5897. doi: 10.1128/JB.00745-10 20870771 10.1128/JB.00745-10 PMC2976465

[55] He K, Marden JN, Quardokus EM, Bauer CE. Phosphate Flow between Hybrid Histidine Kinases CheA3 and CheS3 Controls Rhodospirillum centenum Cyst Formation. PLoS Genet. 2013;9: e1004002 doi: 10.1371/journal.pgen.1004002 24367276 10.1371/journal.pgen.1004002 PMC3868531

[56] Wang F-F, Deng C-Y, Cai Z, Wang T, Wang L, Wang X-Z, et al A three-component signalling system fine-tunes expression kinetics of HPPK responsible for folate synthesis by positive feedback loop during stress response of Xanthomonas campestris. Environ Microbiol. 2013; 10.1111/1462-2920.12293 24119200

[57] Jiménez-Pearson M-A, Delany I, Scarlato V, Beier D. Phosphate flow in the chemotactic response system of Helicobacter pylori. Microbiology. 2005;151: 3299–3311. doi: 10.1099/mic.0.28217-0 16207913 10.1099/mic.0.28217-0

[58] Sourjik V, Schmitt R. Phosphotransfer between CheA, CheY1, and CheY2 in the Chemotaxis Signal Transduction Chain of Rhizobium meliloti. Biochemistry (Mosc). 1998;37: 2327–2335. 10.1021/bi972330a 9485379

[59] Chugani SA, Whiteley M, Lee KM, D’Argenio D, Manoil C, Greenberg EP. QscR, a modulator of quorum-sensing signal synthesis and virulence in Pseudomonas aeruginosa. Proc Natl Acad Sci. 2001;98: 2752–2757. doi: 10.1073/pnas.051624298 11226312 10.1073/pnas.051624298 PMC30211

[60] An D, Apidianakis Y, Boechat AL, Baldini RL, Goumnerov BC, Rahme LG. The Pathogenic Properties of a Novel and Conserved Gene Product, KerV, in Proteobacteria. PLOS ONE. 2009;4: e7167 doi: 10.1371/journal.pone.0007167 19779606 10.1371/journal.pone.0007167 PMC2744870

[61] Mulcahy H, Sibley CD, Surette MG, Lewenza S. Drosophila melanogaster as an Animal Model for the Study of Pseudomonas aeruginosa Biofilm Infections In Vivo. PLOS Pathog. 2011;7: e1002299 doi: 10.1371/journal.ppat.1002299 21998591 10.1371/journal.ppat.1002299 PMC3188550

[62] Limmer S, Haller S, Drenkard E, Lee J, Yu S, Kocks C, et al Pseudomonas aeruginosa RhlR is required to neutralize the cellular immune response in a Drosophila melanogaster oral infection model. Proc Natl Acad Sci. 2011;108: 17378–17383. doi: 10.1073/pnas.1114907108 21987808 10.1073/pnas.1114907108 PMC3198323

[63] Korgaonkar A, Trivedi U, Rumbaugh KP, Whiteley M. Community surveillance enhances Pseudomonas aeruginosa virulence during polymicrobial infection. Proc Natl Acad Sci. 2013;110: 1059–1064. doi: 10.1073/pnas.1214550110 23277552 10.1073/pnas.1214550110 PMC3549110

[64] Opota O, Vallet-Gély I, Vincentelli R, Kellenberger C, Iacovache I, Gonzalez MR, et al Monalysin, a novel ß-pore-forming toxin from the Drosophila pathogen Pseudomonas entomophila, contributes to host intestinal damage and lethality. PLoS Pathog. 2011;7: e1002259 doi: 10.1371/journal.ppat.1002259 21980286 10.1371/journal.ppat.1002259 PMC3182943

[65] Georgescu M, Gheorghe I, Curutiu C, Lazar V, Bleotu C, Chifiriuc M-C. Virulence and resistance features of Pseudomonas aeruginosa strains isolated from chronic leg ulcers. BMC Infect Dis. 2016;16: 92 doi: 10.1186/s12879-016-1396-3 27169367 10.1186/s12879-016-1396-3 PMC4890939

[66] Jaffar-Bandjee MC, Lazdunski A, Bally M, Carrère J, Chazalette JP, Galabert C. Production of elastase, exotoxin A, and alkaline protease in sputa during pulmonary exacerbation of cystic fibrosis in patients chronically infected by Pseudomonas aeruginosa. J Clin Microbiol. 1995;33: 924–929. 7790462 10.1128/jcm.33.4.924-929.1995 PMC228069

[67] Döring G, Obernesser H-J, Botzenhart K, Flehmig B, Høiby N, Hofmann A. Proteases of Pseudomonas aeruginosa in Patients with Cystic Fibrosis. J Infect Dis. 1983;147: 744–750. 6404993 10.1093/infdis/147.4.744

[68] Son MS, Matthews WJ, Kang Y, Nguyen DT, Hoang TT. In Vivo Evidence of Pseudomonas aeruginosa Nutrient Acquisition and Pathogenesis in the Lungs of Cystic Fibrosis Patients. Infect Immun. 2007;75: 5313–5324. doi: 10.1128/IAI.01807-06 17724070 10.1128/IAI.01807-06 PMC2168270

