# Supplementary figures and images for: Regulation of acetyl-CoA synthetase transcription by the CrbS/R two-component system is conserved in genetically diverse environmental pathogens
Source: PLoS One. 2017 May 18;12(5):e0177825. doi: 10.1371/journal.pone.0177825 (PMC5436829; doi:10.1371/journal.pone.0177825)

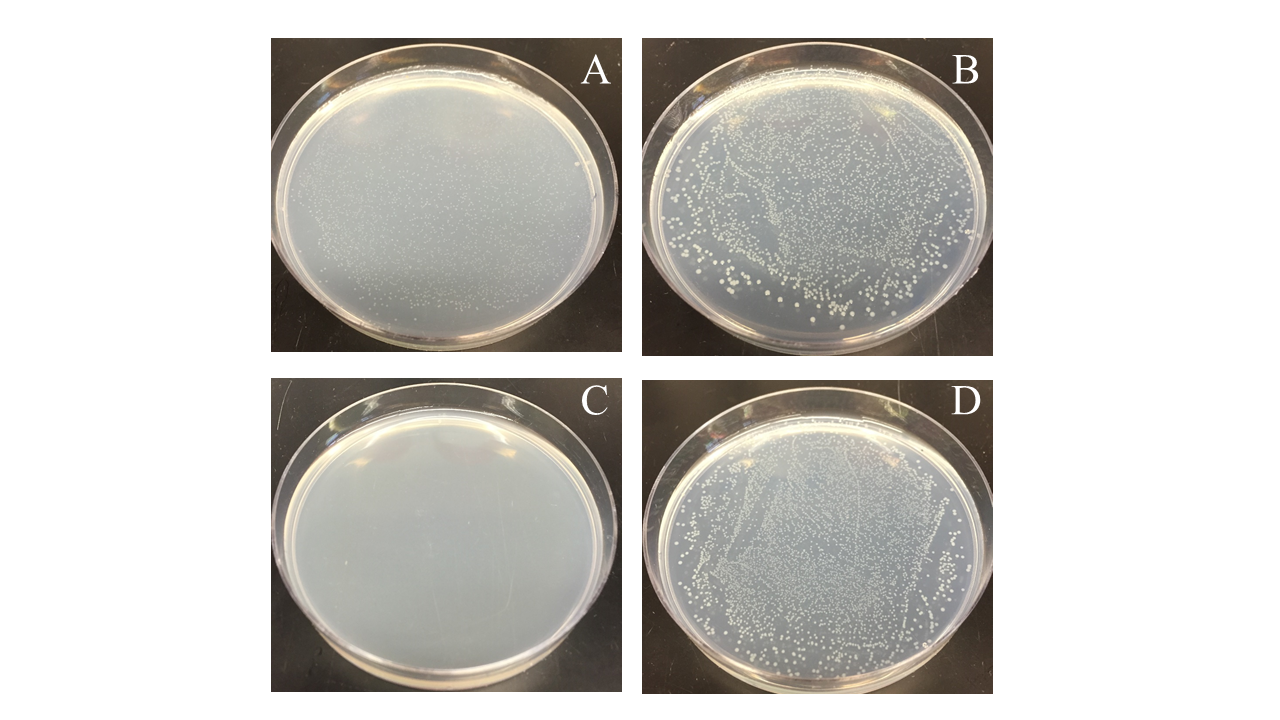

Supplement: S1 Fig — Eight hours postinoculation, 100 μl of culture was spread plated on M63-acetate or M63-acetate/glucose agar supplemented with 1mM IPTG and 30 μg/mL gentamicin as indicated. The strains shown are wild-type Pseudomonas entomophila + pPSV38 on M63 agar with 5mM acetate (A), wild-type Pseudomonas entomophila + pPSV38 on M63 agar with 5mM acetate and 5mM glucose (B), Pseudomonas entomophila ΔacsA + pPSV38 on M63 agar with 5mM acetate(C), and Pseudomonas entomophila ΔacsA + pPSV38 on M63 agar with 5mM acetate and 5mM glucose (D). Note the absence of growth when Pseudomonas entomophila ΔacsA is grown on M63 agar with 5mM acetate as the sole carbon source (C). (TIF) [file pone.0177825.s001.tif]

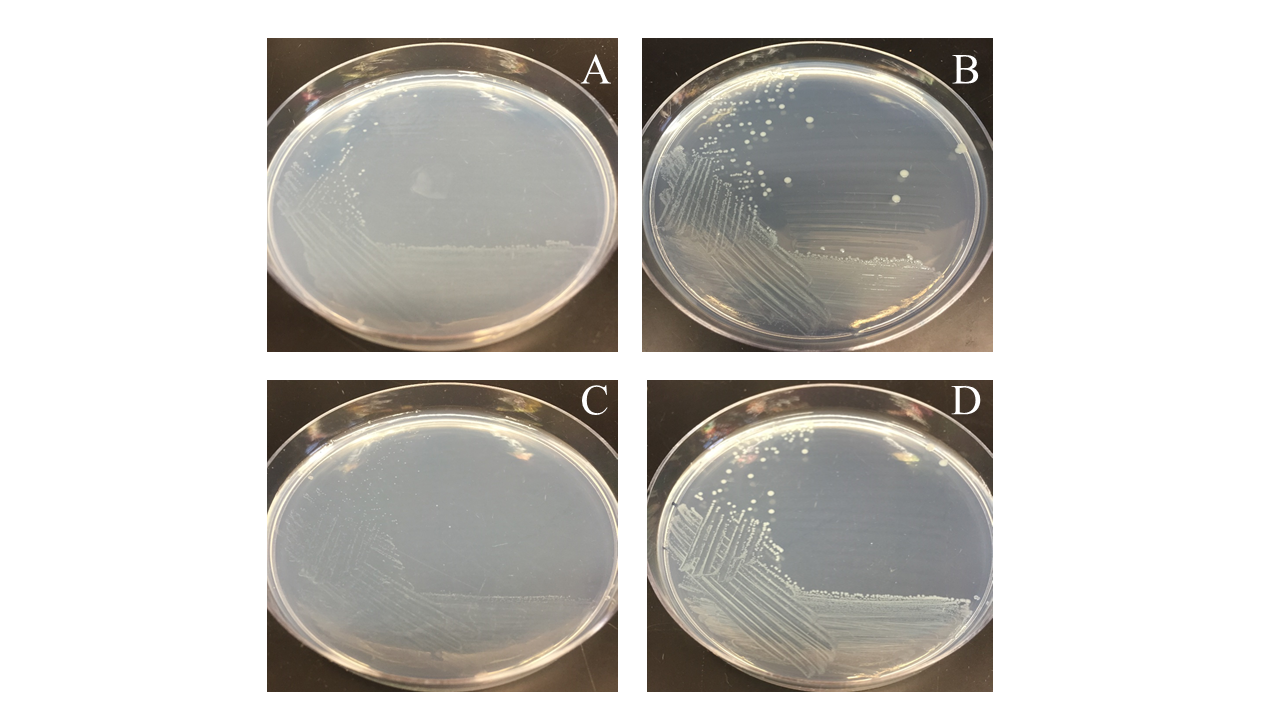

Supplement: S2 Fig — Twenty-four hours postinoculation, 10 μl of culture was quadrant streaked on M63-acetate or M63-acetate/glucose agar supplemented with 1mM IPTG and 30 μg/mL gentamicin as indicated. The strains shown are wild-type Pseudomonas entomophila + pPSV38 on M63 agar with 5mM acetate (A), wild-type Pseudomonas entomophila + pPSV38 on M63 agar with 5mM acetate and 5mM glucose (B), Pseudomonas entomophila ΔacsA + pPSV38 on M63 agar with 5mM acetate (C), and Pseudomonas entomophila ΔacsA + pPSV38 on M63 agar with 5mM acetate and 5mM glucose (D). Note the appearance of suppressor mutants when Pseudomonas entomophila ΔacsA is grown on M63 agar with 5mM acetate as the sole carbon source (C). (TIF) [file pone.0177825.s002.tif]

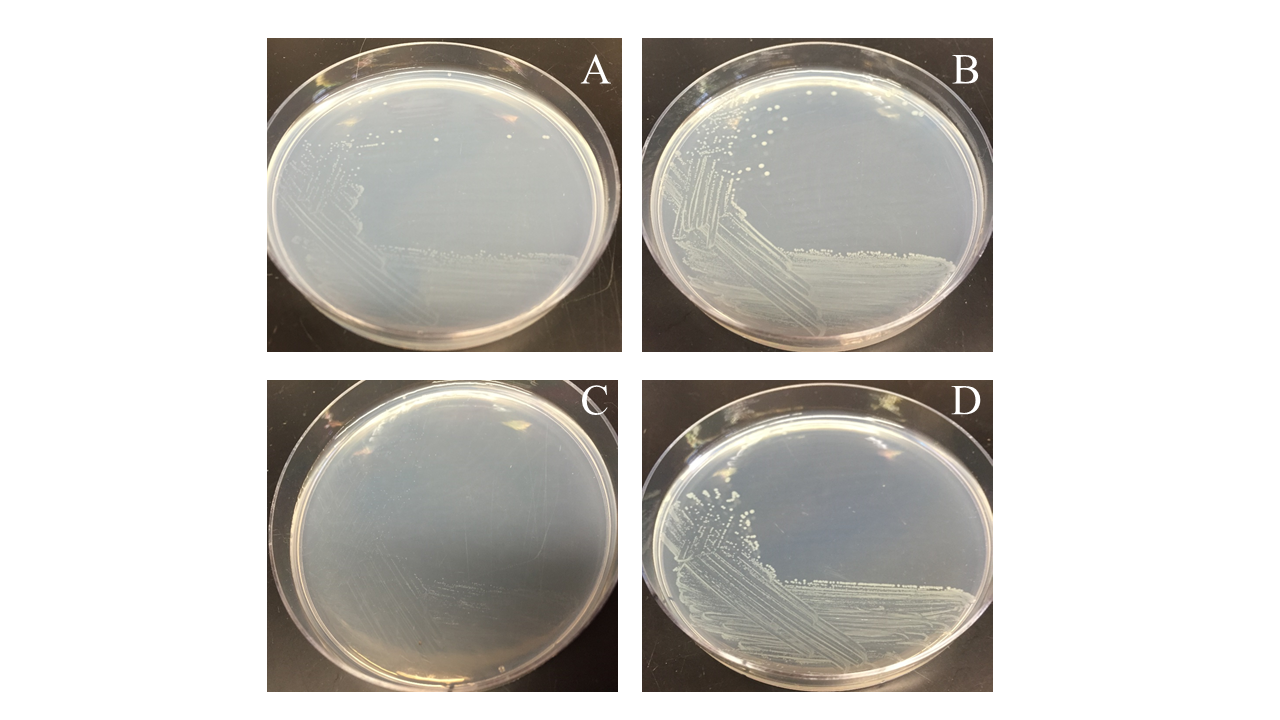

Supplement: S3 Fig — Thirty-six hours postinoculation, 10 μL of culture was quadrant streaked on M63-acetate or M63-acetate/glucose agar supplemented with 1mM IPTG and 30 μg/mL gentamicin, as indicated. The strains shown are wild-type Pseudomonas entomophila + pPSV38 on M63 agar with 5mM acetate (A), wild-type Pseudomonas entomophila + pPSV38 on M63 agar with 5mM acetate and 5mM glucose (B), Pseudomonas entomophila ΔacsA + pPSV38 on M63 agar with 5mM acetate (C), and Pseudomonas entomophila ΔacsA + pPSV38 on M63 agar with 5mM acetate and 5mM glucose (D). Note the appearance of suppressor mutants when Pseudomonas entomophila ΔacsA is grown on M63 agar with 5mM acetate as the sole carbon source (C). (TIF) [file pone.0177825.s003.tif]

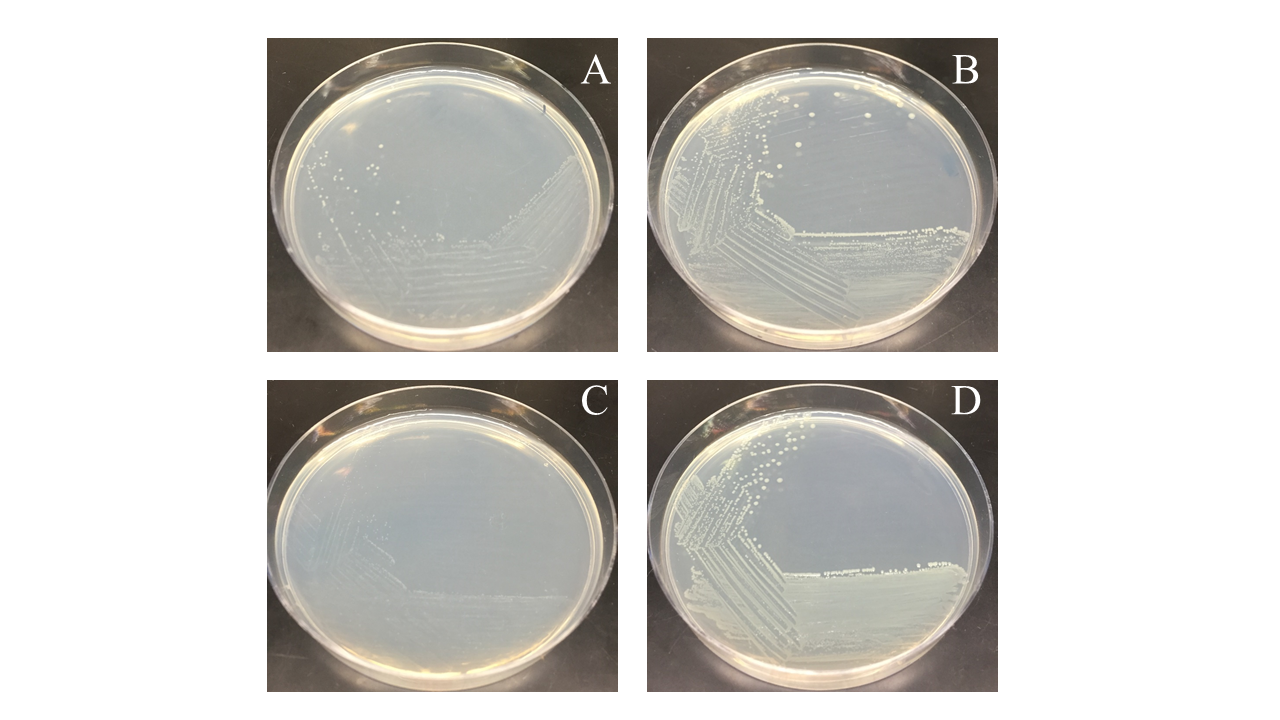

Supplement: S4 Fig — Forty-eight hours postinoculation, 10 μL of culture was quadrant streaked on M63-acetate or M63-acetate/glucose agar supplemented with 1mM IPTG and 30 μg/mL gentamicin as indicated. The strains shown are wild-type Pseudomonas entomophila + pPSV38 on M63 agar with 5mM acetate (A), wild-type Pseudomonas entomophila + pPSV38 on M63 agar with 5mM acetate and 5mM glucose (B), Pseudomonas entomophila ΔacsA + pPSV38 on M63 agar with 5mM acetate (C), and Pseudomonas entomophila ΔacsA + pPSV38 on M63 agar with 5mM acetate and 5mM glucose (D). Note the appearance of suppressor mutants when Pseudomonas entomophila ΔacsA is grown on M63 agar with 5mM acetate as the sole carbon source (C). (TIF) [file pone.0177825.s004.tif]

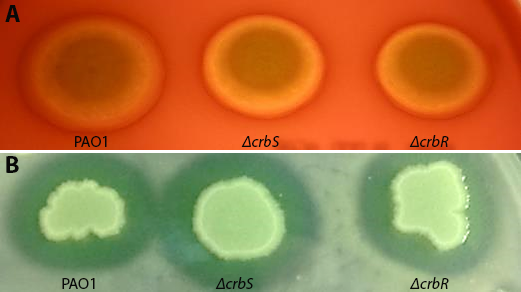

Supplement: S5 Fig — Hemolysin production (A) and AprA protease production (B) of wild-type Pseudomonas aeruginosa PAO1 and mutant strains containing deletions of either crbR or crbS, plated on blood agar or Luria Bertani (Miller) agar + 5% milk, respectively. (TIF) [file pone.0177825.s005.tif]

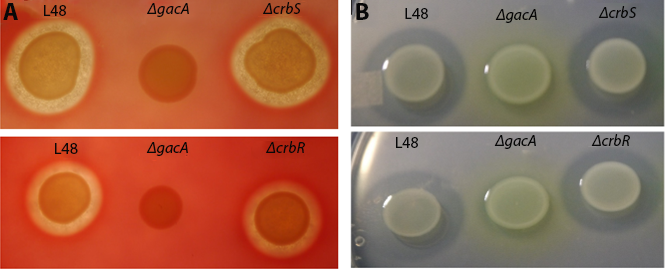

Supplement: S6 Fig — Hemolysin production (A) and AprA protease production (B) of wild-type Pseudomonas entomophila L48 and mutants containing deletions of either gacA, crbR, or crbS. Strains were plated on blood agar for assessing hemolytic activity or modified low salt Luria Bertani (Miller) agar with 5% milk to assay for AprA protease activity. (TIF) [file pone.0177825.s006.tif]
