# Supplementary material for: Regulation of acetyl-CoA synthetase transcription by the CrbS/R two-component system is conserved in genetically diverse environmental pathogens
Source: PLoS One. 2017 May 18;12(5):e0177825. doi: 10.1371/journal.pone.0177825 (PMC5436829; doi:10.1371/journal.pone.0177825)
Supplement: S1 Table — (DOCX) [file pone.0177825.s007.docx]

**S1 Table. Primers used in this study.**

| Primer Name Sequence Description* | | |
| --- | --- | --- |
| AR01_VC0303-P1 | AACTTATGCTGTGGCCCTGT | In-frame deletion of *crbS* in SIO via SOE PCR |
| AR02_VC0303-P2 | **TAACGAGCGGCCGCA**AAACAGCACGCCGAGATAC | In-frame deletion of *crbS* in SIO via SOE PCR; tag in bold |
| AR03_VC0303-P3 | **TGCGGCCGCTCGTTA**CCCATTAAGCCCCTCAAAC | In-frame deletion of *crbS* in SIO via SOE PCR; tag in bold |
| AR04_VC0303-P4 | TTGGCTGGAATGTGTGTGTT | In-frame deletion of *crbS* in SIO via SOE PCR |
| PT47_acs_prom_P1b | GACAACTAGTTTATTGGCAATCGTCCCTTG | Amplification of a 660-bp fragment of the *Acs* promoter, forward, SpeI |
| PT49_acs_prom_P2 | CATTGGATCCAATCCAGTCGACGATTTTGC | Amplification of a 660-bp fragment of the *Acs* promoter, reverse, BamHI |
| PT50_pBBR_F | AATTTCCATTCGCCATTCAG | Amplification of a 488-bp fragment surrounding the SpeI/BamHI cloning sites in pBBR*lux* plasmid, forward |
| PT51_pBBR_R | TTTTCCATCTTTGCCCTACC | Amplification of a 488-bp fragment surrounding the SpeI/BamHI cloning sites in pBBR*lux* plasmid, reverse |
| MMS13_acs_GP1 | ACTCACTATAGGGCCCCCCCCGGCTACCACATTCGTTACG | In-frame deletion of *acs* in SIO via Gibson cloning |
| MMS14_acs_GP2 | GGCAATCAGGCGGTCAACCGG ATAAATATGGGCTTCAC | In-frame deletion of *acs* in SIO via Gibson cloning |
| MMS15_acs_GP3 | GTGAAGCCCATATTTATCCGGTTGACCGCCTGATTGCC | In-frame deletion of *acs* in SIO via Gibson cloning |
| MMS16_acs_GP4 | GGCGGCCGCTCTAGAACGCGG TTAGATTGCAGATGT | In-frame deletion of *acs* in SIO via Gibson cloning |
| ED01_VC2702_P1 | GACAACTAGTATCTCAGGCTTGGTCTGCTC | In-frame deletion of *crbR* in SIO via SOE PCR, SpeI |
| ED02_VC2702_P2 | **TAACGAGCGGCCGCA**GTCCATGACGTCACAGGT | In-frame deletion of *crbR* in SIO via SOE PCR, tag in bold |
| ED03_VC2702_P3SIO | **TGCGGCCGCTCGTTA**AAAAACCGCACGCAAGCAGT | In-frame deletion of *crbR* in SIO via SOE PCR, tag in bold |
| ED05_VC2701_P4 | CATTGGATCCACCATCGGAATTGATGGAGA | In-frame deletion of *crbR* in SIO via SOE PCR, BamHI |
| PT22_VC0303RCD_SIO1 | GGGCCGGGCGATCCGGAAGA | In-frame deletion of *crbS* receiver domain in SIO via SOE PCR |
| PT35_VC0303RCD_TP2C | **TCTCAGTTTGAGGGGCTT**CACACGCAGATGTTGCAGCTCTGAACC | In-frame deletion of *crbS* receiver domain in SIO via SOE PCR, tag in bold |
| PT36_VC0303RCD_TP3C | **AAGCCCCTCAAACTGAGA**GCCTTACTGAATAGTCTGTC | In-frame deletion of *crbS* receiver domain in SIO via SOE PCR, tag in bold |
| PT25_VC0303RCD_4 | GGCTGGAATGTGTGTGTTGG | In-frame deletion of *crbS* receiver domain in SIO via SOE PCR |
| ED80_VC2702REC_P1 | GACAACTAGTATCTCAGGCTTGGTCTGCTC | In-frame deletion of *crbR* receiver domain in SIO via SOE PCR, SpeI |
| ED81_VC2702REC_P2 | TTGATTGAGCGCGGCAATCAGGGTCGAGTCCATGACGTCACA | In-frame deletion of *crbR* receiver domain in SIO via SOE PCR |
| ED82_VC2702REC_P3 | TGTGACGTCATGGACTCGACCCTGATTGCCGCGCTCAATCAA | In-frame deletion of *crbR* receiver domain in SIO via SOE PCR, reverse complement of ED81 |
| ED83_VC2702REC_P4 | CATTGGATCCTTATGGTGTCGTTGGTGGTG | In-frame deletion of *crbR* receiver domain in SIO via SOE PCR, BamHI |
| AEP234_CrbS_H798_P1 | GACAACTAGTGGTGTGTCTTTGGTGACACG | Point mutation of CrbS His-798 to Ala or Gln in SIO via SOE PCR, SpeI |
| AEP235_CrbS_H798A_P2 | GGCTGCATCAAATCcgcACTGACCGCCGCC | Point mutation of CrbS His-798 to Ala in SIO via SOE PCR |
| AEP236_CrbS_H798A_P3 | GGCGGCGGTCAGTgcgGATTTGATGCAGCC | Point mutation of CrbS His-798 to Ala in SIO via SOE PCR |
| AEP237_CrbS_H798_P4 | CATTGAGCTCCAAACGCAAGCGACACTG | Point mutation of CrbS His-798 to Ala or Gln in SIO via SOE PCR, SacI |
| AEP238_CrbS_H798Q_P2 | GGCTGCATCAAATCttgACTGACCGCCGCC | Point mutation of CrbS His-798 to Gln in SIO via SOE PCR |
| AEP239_CrbS_H798Q_P3 | GGCGGCGGTCAGTcaaGATTTGATGCAGCC | Point mutation of CrbS His-798 to Gln in SIO via SOE PCR |
| AEP233_CrbS_HisInt | CATTGGATCCcgaaacgcattagtcaggaa | Sequencing of H798 mutations in SIO genome |
| AEP240_CrbS_D1081A_P1 | GACAACTAGTACGTTCTGAGCGCGAATC | Point mutation of CrbS Asp-1081 to Ala in SIO via SOE PCR, SpeI |
| AEP241_CrbS_D1081A_P2 | CGTTATCCAAGCGGTAcgcAGACAAAATCACATC | Point mutation of CrbS Asp-1081 to Ala in SIO via SOE PCR |
| AEP242_CrbS_D1081A_P3 | GATGTGATTTTGTCTgcgTACCGCTTGGATAACG | Point mutation of CrbS Asp-1081 to Ala in SIO via SOE PCR |
| AEP243_CrbS_D1081A_P4 | CATTGAGCTCGGTTCAAGACCTACGCCAAG | Point mutation of CrbS Asp-1081 to Ala in SIO via SOE PCR, SacI |
| ED47_CrbRdelRCD_intR | GTTGCAAGTAGGCAGCGTAT | Sequencing of D1081A mutation in SIO genome |
| pPSV_PSEEN4122_F1 | ATATGAATTCGAGGAGGATACATATGGCCACATACGAAATCCTGATTG | Cloning *crbR* into the pPSV38 expression vector, EcoRI |
| pPSV_PSEEN4122_R1 | ATATAAGCTTTTAGCTGCCCGAAACCGATTC | Cloning *crbR* into the pPSV38 expression vector, HindIII |
| pPSV_PA3604_F1 | ATATGAATTCGAGGAGGATACATATGGCTTCTTACGAGATCCTGATCG | Cloning *erdR* into the pPSV38 expression vector, EcoRI |
| pPSV_PA3604_R1 | ATATAAGCTTTCAATGAGAAGCGGGAACCGTCTC | Cloning *erdR* into the pPSV38 expression vector, HindIII |
| ΔPSEEN1405_F1 | ATATAAGCTTGCTTCGCCCAGGTGGCATTG | In-frame deletion of *crbS* in *P. entomophila*, HindIII |
| ΔPSEEN1405_R2 | TGAGGCGGTTACGACATGCAAACTCCGGAGCAAAAAATAG | In-frame deletion of *crbS* in *P. entomophila* |
| ΔPSEEN1405_F3 | GCATGTCGTAACCGCCTCATCCGCCAGCGCCCGCTCGA | In-frame deletion of *crbS* in *P. entomophila* |
| ΔPSEEN1405_R4 | ATATGAATTCGAAAGCTACCAGCAGGAGGC | In-frame deletion of *crbS* in *P. entomophila*, EcoRI |
| ΔPSEEN4122_F1 | ATATAAGCTTGTTGATGCATGACACCGAAC | In-frame deletion of *crbR* in *P. entomophila,* HindIII |
| ΔPSEEN4122_R2 | GTTCGGGCACTTAGGCCATCGATCTAGGGATCCTGTTC | In-frame deletion of *crbR* in *P. entomophila* |
| ΔPSEEN4122_F3 | CGATGGCCTAAGTGCCCGAACGTTCACGCTTTTTTGAC | In-frame deletion of *crbR* in *P. entomophila* |
| ΔPSEEN4122_R4 | ATATGGTACCCATCCATTGGCCAAGGTCGG | In-frame deletion of *crbR* in *P. entomophila*, KpnI |
| ΔPSEEN3888_F1 | ATATGGATCCAGGCGGCCCCGGCGATCTTTG | In-frame deletion of *acsA* in *P. entomophila,* BamHI |
| ΔPSEEN3888_R2 | TCCTCATGCGGCCGCCATCTGTGTTACCTCGGTGTAAT | In-frame deletion of *acsA* in *P. entomophila* |
| ΔPSEEN3888_F3 | ACACAGATGGCGGCCGCATGAGGAATGTTCGCCGGCAAGG | In-frame deletion of *acsA* in *P. entomophila* |
| ΔPSEEN3888_R4 | ATATGGTACCGTGATCGACATGGACGACAG | In-frame deletion of *acsA* in *P. entomophila,* KpnI |
| ΔPA3271_F1 | ATATGGATCCCGGTGATGCTGTAGACGAAGG | In-frame deletion of *mxtR* in *P. aeruginosa,* BamHI |
| ΔPA3271_R2 | GAGGCCCGCTCACGACATGCGAACTCCGATGATGTCCC | In-frame deletion of *mxtR* in *P. aeruginosa* |
| ΔPA3271_F3 | CGCATGTCGTGAGCGGGCCTCAGTGACGGCGGATCAGCA | In-frame deletion of *mxtR* in *P. aeruginosa* |
| ΔPA3271_R4 | ATATGAATTCGCGCCGATCGTGCTCCTGCC | In-frame deletion of *mxtR* in *P. aeruginosa*, EcoRI |
| ΔPA3604_F1 | ATATAAGCTTCCGGCCTGGACGATGGCGTC | In-frame deletion of *erdR* in *P. aeruginosa,* HindIII |
| ΔPA3604_R2 | AGCCGTCTCTCAAGCCATGACATGTCCTAGTTTGTTCG | In-frame deletion of *erdR* in *P. aeruginosa* |
| ΔPA3604_F3 | GTCATGGCTTGAGAGACGGCTTAACGCTTTTTTGACCAG | In-frame deletion of *erdR* in *P. aeruginosa* |
| ΔPA3604_R4 | ATATGAATTCCAGAGCTGCCATACCAACAAG | In-frame deletion of *erdR* in *P. aeruginosa,* EcoRI |
| ΔPSEEN1405_REC_F1 | ATATAAGCTTCACTCCTCCGAACGGCTGTT | In-frame deletion of *crbS* receiver domain in *P. entomophila,* HindIII |
| ΔPSEEN1405_REC_R2 | CTGAACCAGGCTCACCTGCAACCCGGCCAGGG | In-frame deletion of *crbS* receiver domain in *P. entomophila* |
| ΔPSEEN1405_REC_F3 | GGGTTGCAGGTGAGCCTGGTTCAGTAACCGCC | In-frame deletion of *crbS* Receiver Domain in *P. entomophila* |
| ΔPSEEN1405_REC_R4 | ATATGAATTCGCAGCGTTTCGCCCAGACCA | In-frame deletion of *crbS* Receiver Domain in *P. entomophila,* EcoRI |
| ΔPSEEN4122_REC_F1 | ATATAAGCTTATACCAAGGTCTGCAGCCAT | In-frame deletion of *crbR* Receiver Domain in *P. entomophila,* HindIII |
| ΔPSEEN4122_REC_R2 | GACCTTTTCGAAGATTTCGTATGTGGCCATCGATC | In-frame deletion of *crbR* Receiver Domain in *P. entomophila* |
| ΔPSEEN4122_REC_F3 | ACATACGAAATCTTCGAAAAGGTCGATGTATCGGC | In-frame deletion of *crbR* Receiver Domain in *P. entomophila* |
| ΔPSEEN4122_REC_R4 | ATATGGTACCCATGCGCCATCACCGCGCCA | In-frame deletion of *crbR* Receiver Domain in *P. entomophila,* KpnI |
| ΔPA2586_gacA_F1 | ATATGGATCCCGACCTTGCGTCATTCC | In frame deletion of *gacA*, BamHI |
| ΔPA2586_gacA_F2 | AATGCGCGACGAGGTGCAGCGTGTAGATGAGCGCC | In frame deletion of *gacA* |
| ΔPA2586_gacA_R3 | CTCATCTACACGCTGCACCTCGTCGCGCATTAGCA | In frame deletion of *gacA* |
| ΔPA2586_gacA_R4 | ATATGGTACCGGCGATCGCCGAAACC | In frame deletion of *gacA*, KpnI |
| ΔPA3271_REC_F1 | GCGCCTGTACTGGATGCGCC | In-frame deletion of *crbS* Receiver Domain in *P. aeruginosa* |
| ΔPA3271_REC_R2 | TCAGCGCAGGCTCACCTGGGCGCCGTTCAGCGCCT | In-frame deletion of *crbS* Receiver Domain in *P. aeruginosa* |
| ΔPA3271_REC_F3 | GGCGCCCAGGTGAGCCTGCGCTGAGCGGGCCTCAG | In-frame deletion of *crbS* Receiver Domain in *P. aeruginosa* |
| ΔPA3271_REC_R4 | GCCGCGGCGGCCGCCTGGGA | In-frame deletion of *crbS* Receiver Domain in *P. aeruginosa* |
| ΔPA3604_REC_F1 | CGCAACCGAAATAGCCGCGC | In-frame deletion of *erdR* Receiver Domain in *P. aeruginosa* |
| ΔPA3604_REC_R2 | GGTGTCCCCGTCGATCTCGTAAGAAGCCATGACAT | In-frame deletion of *erdR* Receiver Domain in *P. aeruginosa* |
| ΔPA3604_REC_F3 | TCTTACGAGATCGACGGGGACACCTGGTGGCCGCC | In-frame deletion of *erdR* Receiver Domain in *P. aeruginosa* |
| ΔPA3604_REC_R4 | TGCTCGCCGCGGCGGGCCTG | In-frame deletion of *erdR* Receiver Domain in *P. aeruginosa* |
| PSEEN3888_RT_F1 | AAAGTGGTGATCACTGCCGACGAA | Real time PCR primer for *acsA* in *P. entomophila* |
| PSEEN3888_RT_R1 | ATGATCTTCTGCACGCTGCTGGTT | Real time PCR primer for *acsA* in *P. entomophila* |
| PA_*acsA*_RT_F1 | TGGTACGACGACCTGATGAA | Real time PCR primer for *acsA* in *P. entomophila* |
| PA_*acsA*_RT_R1 | CCGGAGGTGTAGAGGATGAA | Real time PCR primer for *acsA* in *P. entomophila* |
| clpX_RT_F1 | CGAATGACTGACACCCGTAAT | Real time PCR primer for *clpX* in *P. aeruginosa* and *P. entomophila* |
| clpX_RT_R1 | CGCACTCGTCGCAGATAAA | Real time PCR primer for *clpX* in *P. aeruginosa* and *P. entomophila* |
| PA3234_RT_F1 | GGCATCCTTCCTGGGTATTT | Real time PCR primer for PA3234 (putative acetate transporter) in *P. aeruginosa* |
| PA3234_RT_R1 | ATCATCCTCTTCCTCATCGC | Real time PCR primer for PA3234 (putative acetate transporter) in *P. aeruginosa* |

*****Restriction sites listed in the description are underlined in the oligonucleotide sequence.

**Nucleotides conferring point mutations are in lower case.
