# Supplementary material for: Regulation of acetyl-CoA synthetase transcription by the CrbS/R two-component system is conserved in genetically diverse environmental pathogens
Source: PLoS One. 2017 May 18;12(5):e0177825. doi: 10.1371/journal.pone.0177825 (PMC5436829; doi:10.1371/journal.pone.0177825)
Supplement: S2 Table — Statistical analyses were performed in GraphPad Prism via log-rank analysis. Significance was tested relative to survival of flies that had ingested wild-type bacterial strains. Shaded blocks indicate assays in which the survival curves of the flies differed significantly from those of flies that had ingested wild-type strains (P<0.05). Blocks with bold text indicate assays in which flies died significantly faster than did flies that had ingested the wild-type strains (P<0.05). Blocks without shading or bold text indicate assays in which flies died at a rate that differed insignificantly from flies that ingested wild-type strains (P>0.05). NT, not tested. (DOCX) [file pone.0177825.s008.docx]

**S2 Table. Statistical analyses of *Drosophila* survival curves following ingestion of *Pseudomonas entomophila*, *Pseudomonas aeruginosa,* or *Vibrio cholerae.*** Statistical analyses were performed in GraphPad Prism via log-rank analysis. Significance was tested relative to survival of flies that had ingested wild-type bacterial strains. Shaded blocks indicate assays in which the survival curves of the flies differed significantly from those of flies that had ingested wild-type strains (P<0.05). Blocks with bold text indicate assays in which flies died significantly faster than did flies that had ingested the wild-type strains (P<0.05). Blocks without shading or bold text indicate assays in which flies died at a rate that differed insignificantly from flies that ingested wild-type strains (P>0.05). NT, not tested.

**S2A Table. Survival of *Drosophila* infected with *V. cholerae* SIO strains carrying deletions in the CrbSR signaling pathway as compared to those infected with wild-type SIO.**

|  | **Assay 1** | **Assay 2** | **Assay 3** |
| --- | --- | --- | --- |
| **SIO Δ*crbS*** | P<0.0001 | P<0.0001 | P<0.0001 |
| **SIO Δ*crbR*** | P<0.0001 | P<0.0001 | P<0.0001 |
| **SIO *crbS*ΔREC** | **P=0.0028 (faster)** | P=0.1193 | P=0.3703 |
| **SIO *crbR*ΔREC** | P<0.0001 | P<0.0001 | P<0.0001 |
| **SIO Δ*acs*** | NT | NT | NT |

|  | **Assay 4** | **Assay 5** | **Assay 6** |
| --- | --- | --- | --- |
| **SIO Δ*crbS*** | P<0.0001 | P<0.0001 | P<0.0001 |
| **SIO Δ*crbR*** | P<0.0001 | NT | NT |
| **SIO *crbS*ΔREC** | P=0.3877 | NT | NT |
| **SIO *crbR*ΔREC** | P<0.0001 | NT | NT |
| **SIO Δ*acs*** | P<0.0001 | P<0.0001 | P=0.0001 |

|  | **Assay 7** | **Assay 8** | **Assay 9** |
| --- | --- | --- | --- |
| **SIO Δ*crbS*** | P<0.0001 | P<0.0001 | NT |
| **SIO *crbS*H798A** | P<0.0001 | P<0.0001 | P<0.0001 |
| **SIO *crbS*H798Q** | P<0.0001 | P<0.0001 | P<0.0001 |
| **SIO *crbR*D1081A** | **P=0.0295 (faster)** | **P=0.0025 (faster)** | **P= 0.0354 (faster)** |

**S2B Table. Survival of *Drosophila* infected with *P. entomophila* strains carrying deletions in the CrbSR signaling pathway as compared to those infected with wild-type *P. entomophila*.**

|  | **Assay 1** | **Assay 2** | **Assay 3** |
| --- | --- | --- | --- |
| **Pe Δ*crbS*** | P=0.0409 | P=0.3667 | P=0.077 |
| **Pe Δ*crbR*** | P=0.4352 | P=0.0477 | P=0.9124 |
| **Pe *crbS*ΔREC** | P=0.3319 | P=0.0076 | P=0.008 |
| **Pe *crbR*ΔREC** | P=0.4141 | P=0.1867 | P=0.1521 |
| **Pe Δ*acs*** | P=0.5013 | P=0.0034 | P=0.2572 |

**S2C Table. Survival of *Drosophila* infected with *P. aeruginosa* strains carrying deletions in the CrbSR signaling pathway as compared to those infected with wild-type *P. aeruginosa*.**

|  | **Assay 1** | **Assay 2** | **Assay 3** | **Assay 4** | **Assay 5** | **Assay 6** |
| --- | --- | --- | --- | --- | --- | --- |
| **Pa Δ*mxtR*** | P=0.7755 | P=0.0221 | P=0.8025 | P=0.0135 | P=0.8214 | P=0.2117 |
| **Pa Δ*erdR*** | P=0.1381 | P=0.0165 | P=0.0842 | P=0.1266 | P=0.0663 | P=0.2705 |
